# Spatial organization of pulmonary type 2 inflammation by a macrophage-derived cholesterol metabolite

**DOI:** 10.1101/2025.07.29.666625

**Published:** 2025-08-01

**Authors:** Yufan Zheng, Hannah E. Dobson, Makheni Jean Pierre, Lilly LeBlanc, Chinaemerem U. Onyishi, Dominic P. Golec, Nathan Carrillo, Anshu Deewan, Claudia A. Rivera, Eduard Ansaldo, Eric V. Dang

**Affiliations:** 1 Molecular Mycology and Immunity Section, Laboratory of Host Immunity and Microbiome, Division of Intramural Research, National Institute of Allergy and Infectious Diseases, National Institutes of Health, Bethesda, MD 20892, USA; 2 Immunology Graduate Group, Perelman School of Medicine, University of Pennsylvania, Philadelphia, PA 19104, USA; 3 Department of Microbiology and Immunology, Georgetown University, Washington, DC 20057, USA; 4 Laboratory of Immune System Biology, Division of Intramural Research, National Institute of Allergy and Infectious Diseases, National Institutes of Health, Bethesda, MD 20892, USA; 5 Integrated Data Sciences Section, Research Technologies Branch, Division of Intramural Research, National Institute of Allergy and Infectious Diseases, National Institutes of Health, Bethesda, MD 20892, USA; 6 Metaorganism Immunity Section, Laboratory of Host Immunity and Microbiome, Division of Intramural Research, National Institute of Allergy and Infectious Diseases, National Institutes of Health, Bethesda, MD 20892, USA

## Abstract

Effective pulmonary immunity requires the precise spatial organization of immune cells, yet the mechanisms guiding their intratissue positioning during inflammation remain unclear. Here, we identify a cholesterol-derived chemotactic axis that spatially organizes T helper 2 (T_H_2) cells during fungal-induced pulmonary type 2 inflammation. Inflammation-expanded macrophages expressing cholesterol-25-hydroxylase (CH25H) produce 25-hydroxycholesterol, which is converted into the oxysterol 7α,25-dihydroxycholesterol to attract GPR183-expressing T_H_2 cells into infectious lesions. This T_H_2 positioning suppresses interferon-γ responsiveness in inflammatory Ly6C⁺ macrophages, promoting fungal persistence. Disruption of this axis via T_H_2-specific GPR183 deletion restores type 1 macrophage activation and enhances fungal clearance. Our findings reveal a macrophage-driven, metabolite-based mechanism of immunosuppressive cell positioning in inflamed lung tissue.

Effective immune responses in inflamed tissues require spatial coordination of immune cells. This is particularly critical in the lung, where immune responses must be tightly regulated to prevent excessive inflammation and preserve tissue integrity. However, how immune cells are positioned within inflamed lung tissues, and how such positioning contributes to protective or suppressive outcomes, remains poorly understood. Immune cell positioning has been predominantly characterized in lymphoid organs, where G-protein coupled receptor 183 (GPR183; also known as EBI2) serves as a chemotactic receptor that controls immune cell migration to support dendritic cell homeostasis, T cell-dependent antibody responses, and naïve T cell lymph node entry during inflammation (1–10). GPR183 ligands are cholesterol metabolites called oxysterols, the most potent of which is 7α,25-dihydroxycholesterol (7α,25-HC). Synthesis of 7α,25-HC from cholesterol requires the stepwise action of cholesterol-25-hydroxylase (CH25H) and oxysterol-7α-hydroxylase (CYP7B1) (11–14). At homeostasis, chemotactic 7α,25-HC gradients are generated by non-hematopoietic cells in secondary lymphoid organs, skin, and intestine due to basal constitutive expression of *Ch25h* and *Cyp7b1* by different subsets of stroma (1, 11, 15–19).

Macrophages can induce *Ch25h* expression in response to cytokines such as type I/II interferons and interleukin-4 (IL-4)/IL-13 (14, 20–24). Interferon-induced *Ch25h* has diverse immunomodulatory functions in macrophages (25–32). However, despite being one of the few macrophage IL-4-inducible genes conserved between mice and humans (20, 33), the role of *Ch25h* in type 2 immunity is poorly characterized. Most *Ch25h* functions in macrophages have been attributed to the cell-autonomous activity of 25-HC, as macrophages have low expression of CYP7B1 and thus are unlikely to directly synthesize chemotactic sterols (34). Despite clear evidence that 25-HC can be secreted (28, 34, 35), it remains unknown whether macrophage-derived 25-HC can contribute to 7α,25-HC production in barrier tissues to mediate immune cell migration (15).

GPR183 levels in type 2 lymphocytes were reported to be strongly correlated with asthma outcomes following anti-IL-5 antibody treatment (36), highlighting the potential importance of GPR183 in regulating pulmonary type 2 immunity. During type 2 responses, T helper 2 (T_H_2) cells and/or group 2 innate lymphoid cells (ILC2s) produce cytokines to defend against parasites and to promote tissue repair (37, 38), but can also impair bacterial and fungal clearance (39–41). While ILC2s are tissue-resident and can expand locally upon stimulation, T_H_2 cells are initially primed in the draining lymph nodes and then transmigrate from the blood into the lungs during allergic inflammation in response to CCR4 ligands (CCL17, CCL22) (42, 43). However, after their tissue entry, how T_H_2 cells achieve proper microanatomical positioning to interact with effector cells within the lung parenchyma remains unknown. Here, we show that GPR183 on T_H_2 cells senses macrophage-derived oxysterols to guide positioning towards infection sites and establish local immunosuppressive niches.

## GPR183 is upregulated on T_H_2 during fungal-induced pulmonary type 2 inflammation.

**Fig. 1.**
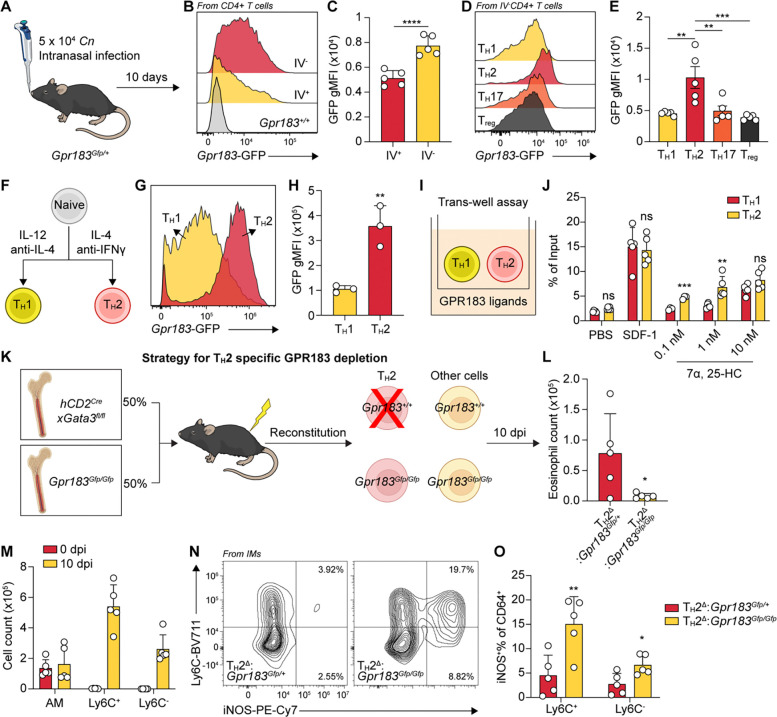
T_H_2 sensing of oxysterols antagonizes iNOS expression in Ly6C^+^ macrophages. (**A**) Schematic of *Cn* intranasal infection model and intravenous (IV) CD45 labeling. (**B**) Representative histogram for *Gpr183*-GFP expression in IV^+^ and IV^−^ CD4^+^ T cells from *Cn*-infected mouse lung tissues at 10 dpi. (**C**) Geometric mean fluorescence intensity (gMFI) of *Gpr183*-GFP in IV^+^ versus IV^−^ CD4^+^ T cells from *Cn*-infected mouse lung tissues at 10 dpi. ^****^P < 0.0001, paired two-tailed Student’s t-test. (**D**) Representative histogram of *Gpr183*-GFP expression across CD4^+^ T cell subsets from *Cn*-infected mouse lung tissues at 10 dpi. (**E**) *Gpr183*-GFP gMFI in CD4^+^ T cell subsets from *Cn*-infected mouse lung tissues at 10 dpi. ^**^P < 0.01 and ^***^P < 0.001 by one-way ANOVA. (**F**) Schematic of *in vitro* activation protocol for naïve T cells using anti-CD3/anti-CD28 beads with indicated cytokines and blocking antibodies. (**G**) Representative histogram of *Gpr183*-GFP expression in *in vitro*-generated T_H_1 and T_H_2 cells. (**H**) Quantification of *Gpr183*-GFP gMFI in *in vitro*-activated T_H_1 and T_H_2 cells. ^**^P < 0.01, unpaired two-tailed Student’s t-test. (**I**) Schematic of trans-well migration assay comparing chemotactic responses of *in vitro* activated T_H_1 and T_H_2 cells to GPR183 ligand, 7α, 25-HC. (**J**) Quantification of T_H_1 and T_H_2 cell migration towards indicated concentrations of 7α, 25-HC and SDF-1. ^**^P < 0.01 and ^***^P < 0.001; ns, not significant; paired two-tailed Student’s t-test. (**K)** Schematic of mixed bone marrow chimera strategy for GPR183 specific depletion in T_H_2 cells. (**L**) Quantification of tissue-resident eosinophils (Live/CD45^+^/IV^−^/CD90.2^−^/ B220^−^/SiglecF^+^/CD11b^+^/SSC-A^hi^) in lungs from *Cn*-infected *Gpr183*^*TH2Δ*^ mice and their controls at 10 dpi. *P < 0.05, unpaired two-tailed Student’s t-test. (**M**) Quantification of lung resident AMs, Ly6C^+^ and Ly6C^−^ IMs at 0 and 10 dpi. (**N**) Representative flow cytometry plots showing iNOS expression in lung CD64^+^ macrophages from *Cn*-infected *Gpr183*^*TH2Δ*^ mice and their controls at 10 dpi. (**O**) Quantification of iNOS expression in lung CD64^+^ macrophages from *Cn*-infected *Gpr183*^*TH2Δ*^ mice and their controls at 10 dpi. *P < 0.05, ^**^P < 0.01, unpaired two-tailed Student’s t-test.

Fungi are potent inducers of type 2 inflammation, with estimates that up to 20% of asthma patients exhibit fungal sensitization (40). Notably, type 2 immunity is detrimental during fungal infections, which pose a growing global health concern (44). Among these, *Cryptococcus neoformans* (*Cn*) was classified as the highest priority fungal pathogen by the World Health Organization in 2022 (45). We infected *Gpr183*^*Gfp/+*^ reporter mice (46) intranasally with 5 × 10^4^ *Cn* (KN99α) to induce pulmonary type 2 inflammation and found that ~60–70 % of lung-tissue resident CD4^+^ T cells expressed GPR183 at 10 days post-infection (dpi), with significantly higher levels than circulating CD4^+^ T cells (Fig. 1, A to C and fig. S1, A to D), implying preferential receptor usage upon tissue entry. Among CD4^+^ T cell subsets, T_H_2 cells had the highest GPR183 expression compared to T_H_1, T_H_17, and regulatory T (T_reg_) cells (Fig. 1, D and E). In vitro polarization of naïve T cells confirmed elevated GPR183 in T_H_2 cells (Fig. 1, F to H). In migration assays, T_H_2 cells exhibited greater sensitivity to the GPR183 ligand 7α,25-HC than T_H_1s, displaying robust chemotaxis toward 0.1 and 1 nM concentrations, whereas T_H_1s only migrated at 10 nM concentrations (Fig. 1, I and J). Importantly, both cell types migrated equally towards 50 nM stromal cell-derived factor 1 (SDF-1) (Fig. 1J). These data link GPR183 upregulation in T_H_2s to increased sensitivity to oxysterol gradients, which may contribute to their microanatomical positioning.

## T_H_2 sensing of oxysterols antagonizes iNOS expression in Ly6C^+^ macrophages

To assess the functional role of GPR183 in T_H_2 cells during pulmonary type 2 inflammation, we generated mixed bone marrow chimeras (*hCD2*^*Cre*^*xGata3*^*fl/fl*^:*GPR183*^*Gfp/Gfp*^), where T_H_2 cells could only derive from *GPR183*^*Gfp/Gfp*^ bone marrow donors because *hCD2*^*Cre*^*xGata3*^*fl/fl*^ mice have a selective T_H_2 deficiency (47), thus resulting in T_H_2 cell-specific GFP-labeled GPR183 deletion (*Gpr183*^*TH2Δ*^) (Fig. 1K). At 10 dpi with *Cn*, *Gpr183*^*TH2Δ*^ mice showed reduced eosinophilia (Fig. 1L). Compared to control mice (*hCD2*^*Cre*^*xGata3*^*fl/fl*^:*GPR183*^*Gfp/+*^), *Gpr183*^*TH2Δ*^ mice showed dramatic increase in inducible nitric synthase (iNOS) expression, particularly in infection-expanded Ly6C^+^ macrophages (Fig. 1, M to O and fig. S1, E and F), suggesting that GPR183 is required on T_H_2 cells to prevent macrophage IFNγ responsiveness. *In vitro*, STAT6 can inhibit IFNγ responsiveness in macrophages by repressing inflammatory gene enhancers (48, 49). However, arginase-1 (ARG1) expression in macrophages was unaltered (fig. S1G), indicating unchanged STAT6 activation. When competed with wildtype macrophages in the same host by mixed bone marrow chimeras, although STAT6-deficient macrophages expressed slightly higher iNOS than wildtype mixing partners, they could not induce iNOS to the same extent as observed in *Gpr183*^*TH2Δ*^ mice (fig. S1, H and I), suggesting only a partial contribution of STAT6 to the suppressed IFNγ responsiveness.

## Lung macrophages establish GPR183 chemotactic gradients via *Ch25h* induction.

**Fig. 2.**
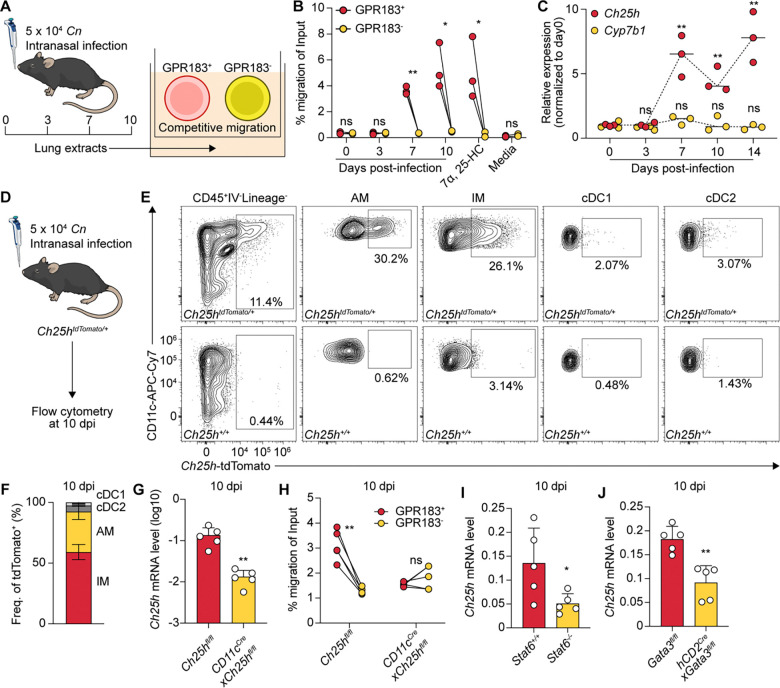
Lung macrophages establish GPR183 chemotactic gradients via *Ch25h* induction. (**A**) Schematic of trans-well migration assay used to assess GPR183-dependent chemotaxis in lung tissue extracts. (**B**) Quantification of migration activity of GPR183^+^ versus GPR183^−^ M12 cells in response to lung extracts collected at indicated time points, 1 nM 7α, 25-HC, or migration media alone. *P < 0.05, ^**^P < 0.01; ns, not significant; paired two-tailed Student’s t-test. (**C**) Relative mRNA levels of *Ch25h* and *Cyp7b1* in lung tissues over time following infection. Expression values were normalized to day 0 to show fold induction. ^**^P < 0.01; ns, not significant; compared to the expression at day 0 by unpaired two-tailed Student’s t-test. (**D**) Schematic of *Ch25h*-tdTomato reporter experiments. (**E**) Representative flow cytometry plots of *Ch25h*-tdTomato expression across indicated immune cell populations from *Cn*-infected mouse lung tissues at 10 dpi. (**F**) Proportional contribution of each indicated population to total tdTomato^+^ (*Ch25h*-expressing) cells in *Cn*-infected mouse lung tissues at 10 dpi. (**G**) Relative mRNA levels of *Ch25h* to *Rplp0* in lung tissues from *Cn*-infected *CD11c*^*Cre*^*xCh25h*^*fl/fl*^ mice or their controls at 10 dpi. ^**^P < 0.01, unpaired two-tailed Student’s t-test. (**H**) Quantification of GPR183^+^ versus GPR183^−^ M12 cell migration towards lung extracts from *CD11c*^*Cre*^*xCh25h*^*fl/fl*^ mice and littermate controls at 10 dpi. ^**^P < 0.01; ns, not significant; paired two-tailed Student’s t-test. (**I**) Relative mRNA levels of *Ch25h* to *Rplp0* in lung tissues from *Cn*-infected STAT6-deficient mice or their controls at 10 dpi. (**J**) Relative mRNA levels of *Ch25h* to *Rplp0* in lung tissues from *Cn*-infected T_H_2-deficient mice or their controls at 10 dpi.

We utilized a transwell migration assay to assess GPR183 ligand activity in lung tissues, comparing the migration of M12 cells (B lymphoma cell line) transduced with MSCV-*Gpr183*-IRES-*Gfp* versus non-transduced cells towards lung extracts (Fig. 2A). GPR183^+^ cells exhibited significantly greater migration than GPR183^−^ cells towards lung extracts from infected mice at 7 and 10 dpi, but not at 0 or 3 dpi (Fig. 2B), arguing that GPR183 ligand is synthesized *de novo* during infection. While 7α,25-HC is the most potent GPR183 ligand, there is also reported receptor activity for 7α,24-HC (synthesized by CYP46A1 and CYP39A1) and 7α,27-HC (synthesized by CYP27A1 and CYP7B1) (1, 13, 14, 17). Of the three enzymes catalyzing the initial cholesterol side chain hydroxylation step, *Ch25h* showed the most robust and sustained induction during infection (Fig. 2C), whereas *Cyp27a1* expression was undetectable and *Cyp46a1* was only transiently induced at 7 dpi (fig. S2A), albeit at ~1000-fold lower absolute levels than *Ch25h (*fig. S2B). The expression of *Cyp7b1*, responsible for the 7α-hydroxylation step downstream of CH25H and CYP27A1, remained largely unchanged (Fig. 2C). These data suggest that *Ch25h* induction is a key driver of the increased GPR183 ligand activity observed in the lung during infection.

Given the known inducible expression of *Ch25h* in macrophages, we hypothesized that lung macrophages might represent the primary source of 25-HC during type 2 inflammation. Supporting this hypothesis, flow cytometry analysis of *Ch25h*^*tdTomato/+*^ reporter mice (9) infected with *Cn* for 10 days revealed that among all hematopoietic cells, *Ch25h* expression was restricted exclusively to CD11c^+^ macrophages including both alveolar macrophages (AMs) and interstitial macrophages (IMs) but not to classical dendritic cells (cDC1s and cDC2s)(Fig. 2, D to F; fig. S2C). To further validate this, we generated *CD11c*^*Cre*^x*Ch25h*^*fl/fl*^ mice to selectively delete *Ch25h* in CD11c^+^ cells. In these mice, *Ch25h* expression in whole lung was reduced by approximately 10-fold compared to littermate controls at 10 dpi (Fig. 2G), approaching or falling below baseline levels, suggesting that CD11c^+^ macrophages are the major source of *Ch25h* in the lung. Consistently, day 10 post-infection lung extracts from these mice failed to promote GPR183^+^ cell migration, in contrast to extracts from *Ch25h*^*fl/fl*^ mice (Fig. 2H), arguing that macrophages are the principal driver of GPR183 dependent chemotaxis by providing 25-HC that is converted into 7α,25-HC in the lung during infection.

Notably, *Ch25h* induction was reduced in *Stat6*^−*/*−^ and T_H_2-deficient mice, suggesting a partial dependence on T_H_2 cell-mediated type 2 signaling (Fig. 2, I and J). Consistent with this, IL-4 modestly induced *Ch25h* expression in bone marrow-derived macrophages (BMDMs), and this induction was markedly enhanced by *Cn* stimulation (fig. S2D), likely due to upregulation of IL-4 receptor expression (50). Consistent with published observations that the SREBP pathway is important for macrophage alternative activation, as 25-HC inhibits SREBP via INSIG1 binding (51, 52), *Ch25h*-deficient BMDMs and PEMs showed an elevated ARG1 expression compared to wildtype controls (fig. S2, E to M), supporting a role for *Ch25h* as a negative regulator of macrophage responses to type 2 cytokines. Thus, *Ch25h* is unlikely to promote type 2 immunity by enhancing alternative activation of macrophages.

## Single-cell RNA sequencing reveals distinct transcriptional programs of *Ch25h*-expressing macrophages

Tissue macrophages originate from either embryonic progenitors or adult granulocyte-monocyte progenitor (GMP)-derived monocytes (53). At 10 dpi, the majority of Ly6C^+^ macrophages – including ~80% of Ly6C^+^ IMs and ~90% of Ly6C^+^ AMs – were *Ms4a3*-tdTomato^+^, indicating a monocyte origin (53). In *Ch25h*^*tdTomato/+*^ reporter mice at the same time point, *Ch25h* expression was significantly enriched in Ly6C^−^ IMs and AM compared to the more monocyte-derived Ly6C^+^ population. These findings suggest that *Ch25h* induction is influenced by macrophage ontogeny, with preferential expression in subsets less associated with recent monocyte recruitment.

**Fig. 3.**
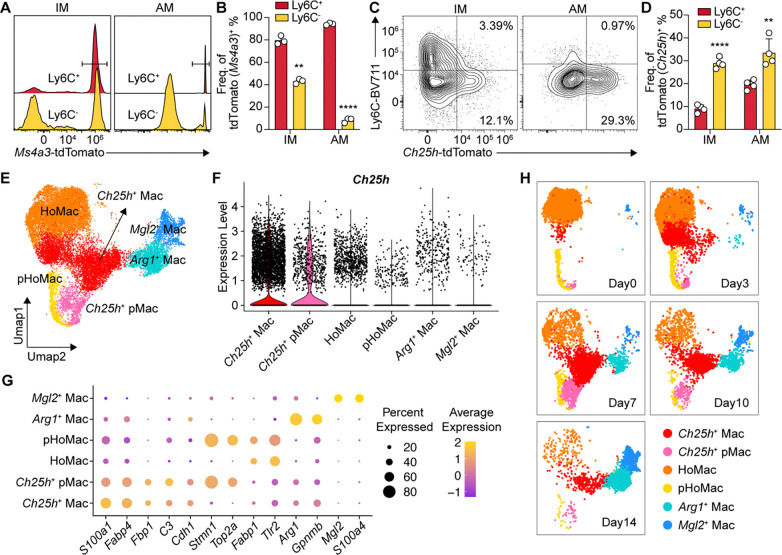
Single-cell RNA sequencing reveals distinct transcriptional programs of *Ch25h*-expressing macrophages (**A**) Representative histogram of *Ms4a3*-tdTomato expression in Ly6C^+^ versus Ly6C^−^ IMs and AMs from *Cn*-infected mouse lungs at 10 dpi. (**B**) Ms4a3^+^ frequency of Ly6C^+^ versus Ly6C^−^ IMs and AMs from *Cn*-infected mouse lungs at 10 dpi. ^****^P < 0.0001 by one-way ANOVA. (**C**) Representative flow cytometry plot of *Ch25h*-tdTomato expression in Ly6C^+^ versus Ly6C^−^ IMs and AMs from *Cn*-infected mouse lungs at 10 dpi. (**D**) Frequency of *Ch25h*^*+*^ within Ly6C^+^ versus Ly6C^−^ IMs and AMs from *Cn*-infected mouse lungs at 10 dpi. ^****^P < 0.0001; paired two-tailed Student’s t-test. (**E**) UMAP visualization of annotated macrophage subsets from combined single-cell RNA sequencing replicates (21,069 cells from 15 mice; 3 mice per time point). (**F**) Violin plot showing *Ch25h* expression across indicated macrophage subsets. (**G**) Top differentially expressed genes within each macrophage population. (**H**) UMAP visualization of macrophage subset distribution across individual time points.

To better characterize *Ch25h*-expressing macrophages, we performed single-cell RNA sequencing (scRNA-seq) on sorted Ly6C^−^ lung macrophages (MertK^+^CD64^+^Ly6C^−^) from 15 mice across five time points post-*Cn* infection (n=3/time point; fig. S3A). Initial clustering revealed 8 macrophage populations (Mac1–8), including three *Ch25h*-high populations (Mac3, 4, and 6) with *Ch25h* ranking among their top 10 differential expressed genes (fig. S3B). Mac3 and Mac4 shared similar transcriptional profiles and were combined as *Ch25h*^*+*^ macrophages (*Ch25h*^*+*^ Mac). Mac6 expressed high levels of proliferation markers (*Stmn1*, *Top2a*, and *Mki67*) and were annotated as *Ch25h*^*+*^ proliferating macrophages (*Ch25h*^*+*^ pMac) (Fig. 3E and F; fig. S3B). Homeostatic clusters (Mac1, 2, and 5) were categorized into homeostatic macrophages (HoMac) and proliferating homeostatic macrophages (pHoMac) based on proliferative gene signatures (Fig. 3E). Mac7 and Mac8 were classified by their defining markers, *Arg1* and *Mgl2*, respectively (Fig. 3E). *Ch25h*^*+*^ Mac showed elevated expression of *Fabp4* and *Fbp1*, indicating potential metabolic reprogramming, along with *Cdh1*, a marker of alternative activation and epithelioid transformation (54, 55) (Fig. 3G). Their abundance peaked at 7 dpi and 10 dpi (Fig. 3H), consistent with peak GPR183-dependent migratory activity.

Together, these data identify a subset of specialized CD11c^+^ Ly6C^−^ macrophages as the dominant source of inflammation-induced *Ch25h* expression, establishing chemotaxis in lung tissues to promote GPR183^+^ cell migration.

## GPR183 is dispensable for T cell recruitment or differentiation.

As GPR183 has been reported to promote eosinophil and macrophage entry to lung tissues in inflammatory settings (56–60), we asked whether GPR183 controlled T_H_2 lung entry during fungal infection. At 10 dpi, *Gpr183*^*TH2Δ*^ mice showed no difference in T_H_2 numbers compared to controls (fig. S4A), indicating that GPR183 is dispensable for T_H_2 recruitment. We additionally generated mixed bone marrow chimeras using CD45.1^+^ wildtype and CD45.2^+^ *Gpr183*^*Gfp/+*^ or *Gpr183*^*Gfp/Gfp*^ donors to assess the contribution of GPR183 to tissue entry. At 10 dpi, no differences were observed between the contribution of CD45.2^+^ cells to tissue resident and circulating CD4^+^ T cells, or to lung resident CD4^+^ T cell subsets (fig. S4, B to F), further supporting a recruitment-independent role for GPR183.

As GPR183 has been described to regulate CD4^+^ T cell priming in lymph nodes during ovalbumin immunization (16), we also assessed T cell differentiation in GPR183-knockout mixed bone marrow chimeras in both lung tissues and lung-draining mediastinal lymph nodes. At 10 dpi, GATA3, T-bet, and RORγT expression, as well as cytokine production upon restimulation, were equivalent in lung-resident CD4^+^ T cells (fig. S4, G to O) and no differences were found in GATA3 or T-bet expression in CD4^+^ T cells in lymph nodes (fig. S4, P and Q), indicating that GPR183 does not affect CD4^+^ T cell differentiation in the setting of fungal infection.

To test whether any effects of GPR183 on T_H_2 recruitment or differentiation were being masked in the polyclonal response, we generated *Cn* chitin deacetylase 2(CDA2)-specific TCR transgenic (CnT II) mice (61, 62) that were either *Gpr183*^*Gfp/+*^ (CD45.1) or *GPR183*^*Gfp/Gfp*^ (CD45.1/2) and performed competitive adoptive CD4^+^ T cell transfers into *Ch25h*^*fl/fl*^ or *CD11c*^*Cre*^x*Ch25h*^*fl/fl*^ mice (CD45.2) (fig. S5, A and B). At 10 dpi, the ratio of transferred *Gpr183*^*Gfp/+*^ to *GPR183*^*Gfp/Gfp*^ T cells in the lung was identical between *Ch25h*^*fl/fl*^ and *CD11c*^*Cre*^x*Ch25h*^*fl/fl*^ mice (fig. S5C), and the GATA3^hi^ % was equivalent between *Gpr183*^*Gfp/+*^ and *GPR183*^*Gfp/Gfp*^ transferred FOXP3^−^CD4^+^ T cells (fig. S5D) in both recipients, further supporting a dispensable role for GPR183 in fungal antigen-specific CD4^+^ T cell recruitment and T_H_2 differentiation.

## GPR183 orients T cells towards fungal granulomatous lesions.

Given that GPR183 deficiency did not affect T cell recruitment or differentiation, we hypothesized that it regulates intrapulmonary positioning. As wildtype KN99α *Cn* infection typically causes diffuse lung infection that makes it difficult to resolve positioning differences, we utilized a granuloma-forming strain (41, 63), *Cn gcs1Δ* (5 × 10^4^ intranasal infection), to spatially observe immune cell positioning within organized infectious lesions. We first confirmed no T cell recruitment or differentiation effects by GPR183 in this model at 21 dpi (fig. S6, A to C), when the mice had the highest T_H_2 cell numbers within the infection time course (41).

**Fig. 4.**
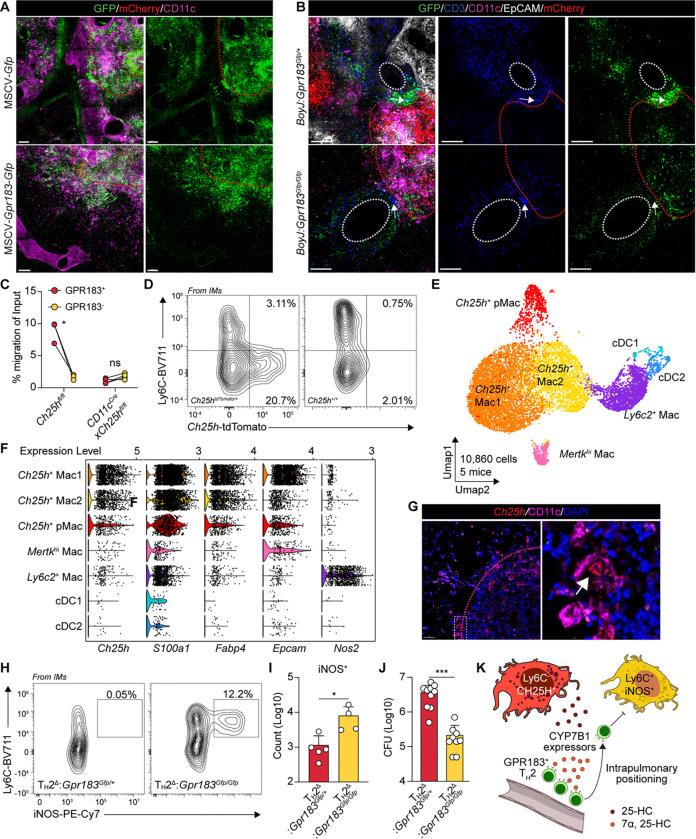
GPR183 guides intrapulmonary positioning of T_H_2 cells to antagonize fungal clearance within granulomatous lesions. (**A**) Representative full-projection confocal images of 500 μm thick lung sections from bone marrow chimeras overexpressing MSCV-*Gfp* or MSCV-*Gpr183*-*Gfp* from *Cn gcs1Δ*-infected at 35 dpi. (**B**) Representative full-projection confocal images of 500 μm thick lung sections from *Cn gcs1Δ*-infected *Gpr183*-knockout mixed bone marrow chimeras or their controls at 35 dpi. (**C**) Quantification of migration activity of GPR183^+^ versus GPR183^−^ M12 cells in response to lung extracts collected from *CD11c*^*Cre*^*xCh25h*^*fl/fl*^ mice or their controls at 35 dpi. *P < 0.05; ns, not significant; paired two-tailed Student’s t-test. (**D**) Representative flow cytometry plots of *Ch25h*-tdTomato expression in lung CD64^+^ macrophages at 35 dpi. (**E**) UMAP visualization of annotated myeloid cell subsets from merged scRNA-seq replicates (10,860 cells from 5 mice at 35 dpi). (**F**) Expression patterns of selected genes across indicated myeloid subsets. (**G**) Representative imaging of *Ch25h* transcripts detected by RNAscope, co-stained with CD11c by immunofluorescence on thin sections of *Cn gcs1Δ*-infected mouse lung tissues at 35 dpi. (**H**) Representative flow cytometry plots showing iNOS expression in CD64^+^ lung macrophages from *Cn gcs1Δ*-infected *Gpr183*^*TH2Δ*^ mice and their controls at 21 dpi. (**I**) Quantification of iNOS^+^ lung macrophages in *Cn gcs1Δ*-infected *Gpr183*^*TH2Δ*^ mice and their controls at 21 dpi. (**J**) Pulmonary fungal burden in *Cn gcs1Δ*-infected *Gpr183*^*TH2Δ*^ mice and their controls at 21 dpi. CFU, colony forming unit. (**K**) Schematic for the proposed model. During inflammation, Ly6C^+^ macrophages induce *Ch25h* expression, leading to the production of oxysterols that establish a GPR183-mediated chemotaxis. This guides T_H_2 intrapulmonary positioning, which in turn antagonize local IFN-γ responses.

To test whether GPR183 regulates parenchymal immune cell positioning, we overexpressed GPR183 in hematopoietic cells by transducing a MSCV-*GPR183*-*Gfp* retroviral vector into bone marrow, which was then transplanted into irradiated recipients. At 35 dpi, the peak of pulmonary fungal load when organized structures start being observed, by thick tissue confocal imaging, we found that GPR183-overexpressing cells were all localized with the granulomatous area at 35 dpi (defined by CD11c staining and *Cn* mCherry signals), whereas a large portion of control GFP-transduced immune cells were still around vasculature (defined by autofluorescence) (Fig. 4A). Quantitative spatial analysis showed a bimodal distribution of control GFP^+^ cells: one peak near the vasculature and another one at the infection site represented by high density of mCherry signals, while GPR183-overexpressing cells only displayed a single peak colocalized with mCherry peak-infection sites (fig. S6D), demonstrating that GPR183 overexpression could force immune cell migration into granulomatous lesions.

Next, we performed similar thick section imaging on lung tissues derived from mixed bone marrow chimeras (*BoyJ*:*Gpr183*^*Gfp/+*^ and *BoyJ*:*Gpr183*^*Gfp/Gfp*^) with CD3 co-staining to directly ask whether oxysterols could direct T cell positioning through GPR183. We found that generally, there was one group of CD3^+^ T cells around vasculature and another group aggregating towards granulomatous lesions at 35 dpi (Fig. 4B). In *BoyJ*:*Gpr183*^*Gfp/+*^ chimeras, GFP^+^ GPR183-expressing T cells were oriented towards the granulomatous lesions, which was not observed in *BoyJ*:*Gpr183*^*Gfp/Gfp*^ chimeras, where the GFP^+^ GPR183-deficient cells were distributed uniformly around the vasculature (fig. S6E). These data, together with the GPR183-overexpression data, demonstrate that T cells use GPR183 to orient towards granulomatous lesions within the inflamed lung after tissue entry.

## Granuloma-associated macrophages express *Ch25h* during persistent fungal infection.

CH25H expression is found within human and mouse mycobacterial granulomas (64). As we observed macrophage-derived oxysterols contributing to GPR183 ligand generation during acute infection, we asked whether this also occurs within fungal granulomatous infection. Migration assays showed an increase in GPR183 ligand activity from lung extracts from *Cn gcs1Δ* infected mice across a time course (fig. S7A). *Ch25h* specific deletion in CD11c^+^ cells also abolished the GPR183-dependent migration activity at 35 dpi (Fig. 4C), suggesting a similar group of macrophages expressed *Ch25h* during fungal granulomatous infection that was found during acute infection. Consistently, we found the same enrichment of *Ch25h* signal in CD11c^+^ Ly6C^−^ macrophages at 35 dpi using *Ch25h*^*tdTomato/+*^ reporter mice (Fig. 4D and fig. S7B).

We next performed scRNA-seq on broad myeloid cells (CD64^+^MHCII^+^CD11c^+^ ) to better characterize *Ch25h*^*+*^ macrophages during fungal granulomatous infection at 35 dpi. Initial clustering identified seven populations (C1 to C7) (fig. S7C). Two clusters (C6 and C7) expressed high *Zbtb46* and were annotated as cDC1 (*Itgae*^*+*^) and cDC2 (*Mgl2*^+^). The remaining clusters (C1 to C5) expressed *Mertk*, consistent with macrophage identity. C4 with the highest *Mertk* expression, was named *Mertk*^hi^ Mac. Although C5 had lower *Mertk*, it expressed *Mafb*, a transcription factor favoring macrophage over DC differentiation, and was named *Ly6c2*^+^ Mac (fig. S7D).

*Ch25h* expression was enriched in macrophage clusters-C1, C2, and C3 (fig. S7D). *Ly6c2*^+^ Mac did not express *Ch25h*, consistent with the reporter data. C1, expressing proliferative markers (fig. S7D), was named as *Ch25h*^+^ pMac, while C2 and C3 were subsequently named as *Ch25h*^+^ Mac1 and *Ch25h*^+^ Mac2 (Fig. 4E). Notably, *S100a1* and *Fabp4* were consistently co-expressed with *Ch25h* within these clusters. While *Cdh1* expression was low compared to acute infection, *Ch25h*^+^ Mac1 and pMac expressed *Epcam*, an epithelial marker associated with macrophage epithelioid transformation (Fig. 4F). To localize *Ch25h* spatially, we performed *Ch25h* RNAscope *in situ* hybridization with CD11c co-staining and found that *Ch25h* signals were enriched in large-body CD11c^+^ cells at the granuloma border (Fig. 4G).

## T_H_2 sensing of oxysterols antagonizes fungal clearance within granulomatous lesions.

Consistent with wildtype KN99α *Cn* infection, during KN99α *Cn gcs1Δ* infection, *Gpr183*^*TH2Δ*^ mice showed the same increase in iNOS expression in Ly6C^+^ macrophages while ARG1 still remained unchanged (Fig. 4, H and I; fig. S7E) at 21 dpi. Notably, scRNA-seq showed an enrichment of *Nos2* in *Ly6c2*^+^*Mafb*^*+*^Mac in wildtype mice (Fig. 4F), suggesting Ly6C^+^ macrophages were the main IFNγ signaling responders. A protective role for IFNγ has been described in chronic *Cn* infection (41, 65–70). Therefore, we asked whether the increase of IFNγ responsiveness corresponded to a lower pulmonary fungal load. Indeed, *Gpr183*^*TH2Δ*^ mice showed significantly lower pulmonary fungal burden than controls at both 21 and 35 dpi (Fig. 4J and fig. S7F).

In summary, our data suggested a model where Ly6C^−^ *Ch25h*-expressing macrophages produce 25-HC to establish chemotactic gradients that guide T_H_2 positioning towards infectious lesions, thus suppressing iNOS production in Ly6C^+^ macrophages and promoting persistent fungal infection (Fig. 4K).

## Discussion

This study identifies a critical mechanism for T_H_2 positioning within the inflamed lung. Although chemokines are known to regulate cell trafficking from the blood into the lung parenchyma, identification of chemotactic receptors that uncouple recruitment from post-entry positioning has been challenging. Here, we provide direct evidence that spatial localization, independent of recruitment, is a distinct and essential layer of immunoregulation during pulmonary type 2 inflammation. In inflamed lung tissues, GPR183, a well-established chemotactic receptor in lymphoid organs, guides recruited T cells towards infection sites, thereby shaping local immune responses.

Langerhans cells have been shown to partially contribute to GPR183-dependent naïve lymphocyte recruitment into inflamed lymph nodes (7), suggesting that inflammation-dependent *Ch25h* expression in myeloid cells can contribute to tissue entry. Here, we identify CD11c^+^Ly6C^−^ lung macrophages that emerge during pulmonary fungal infection as major 25-HC producers establishing GPR183-dependent chemotaxis to guide T_H_2 positioning. Notably, *Cyp7b1* does not significantly increase upon infection, suggesting that *Ch25h* induction in macrophages is rate-limiting for production of chemotactic oxysterols in the lung. Distinct from what has been shown with other cell types (5–9, 71), we did not observe a requirement for GPR183 in T cell tissue entry, indicating either redundancy or lack of function in this process. We found that STAT6- or T_H_2-deficient mice have reduced *Ch25h* expression at the whole lung level, suggesting a contribution from type 2 cytokines to *Ch25h* induction, potentially in cooperation with interferon signaling. *Ch25h*^+^ macrophages display elevated lipid metabolism signatures in scRNA-seq data, which may contribute to *Ch25h* upregulation or 25-HC synthesis. In line with previous findings (72), homeostatic AMs express baseline levels of *Ch25h*, but we observed little GPR183-dependent migration activity in lung extracts from uninfected mice. This implies that AM-derived 25-HC may either have alternative functions or spatial distributions insufficient to establish chemotactic gradients in cooperation with functional *Cyp7b1*^+^ cells, which are likely adventitial fibroblasts based on expression data from different lung cell types (73).

Our findings also offer mechanistic insights into granuloma organization. Beyond cellular recruitment, there must exist spatial cues within tissues that guide immune cell positioning towards granulomatous lesions. We identify a non-redundant role for GPR183-mediated chemotaxis in organizing T_H_2 cells within these microanatomical structures that emerge during chronic fungal infection. Specifically, we show that *Ch25h*^+^ macrophages, expressing markers of epithelioid transformation, guide T_H_2 localization through GPR183. This regulation might be conserved in granulomatous structures across different infectious and non-infectious settings. Supporting this idea, CH25H expression has been reported around human tuberculosis granulomas (64). T lymphocyte cuffs are a common feature of granulomas (74, 75), resembling tertiary lymphoid structures that can support both pathogen clearance and persistence. Our study suggests that granuloma-associated macrophages actively shape this niche by directing T_H_2 positioning, thereby promoting local immunosuppression and pathogen persistence.

Additionally, our study sheds new light on the mechanisms underlying type 1 and type 2 immune competition during mixed inflammation. A tempting hypothesis is that T_H_2 cells outcompete T_H_1 cells for oxysterol gradients to co-localize with Ly6C^+^ macrophages, thereby suppressing local type 1 response. Consistent with this idea, GPR183-deficient T_H_2 cells failed to restrain IFNγ responsiveness in macrophages, despite normal tissue T_H_2 numbers, highlighting the importance of spatial proximity for this regulatory interaction. Importantly, this effect is unlikely to be explained solely by loss of STAT6-dependent competition with STAT1. These findings point to a proximal, spatially constrained crosstalk, where Ly6C^−^ macrophages guide T_H_2 cells to Ly6C^+^ macrophages to potentially ‘shield’ them from T_H_1s, thus preventing iNOS induction.

The potential importance of GPR183 has recently been suggested by a clinical study in asthma patients, which found that the therapeutic efficacy of anti-IL-5 treatment correlated with reduced GPR183 expression on type 2 lymphocytes (36). Although we did not observe a lung entry effect for GPR183 on T_H_2 cells, our findings demonstrate that GPR183-mediated chemotaxis is essential for their intra-parenchymal migration to inflammation sites, thereby shaping the local immune responses. From a translational perspective, manipulating this spatial axis could offer therapeutic strategies for diseases involving type 1/2 immune imbalances.

## Supplementary Material

Supplement 1

## Data Availability

All source data are available upon request.

## References

[1] Lu E., Dang E. V., McDonald J. G., Cyster J. G., Distinct oxysterol requirements for positioning naïve and activated dendritic cells in the spleen. Sci Immunol 2, (2017). 10.1126/sciimmunol.aal5237 PMC5646419 28738017

[2] Li J., Lu E., Yi T., Cyster J. G., EBI2 augments Tfh cell fate by promoting interaction with IL-2-quenching dendritic cells. Nature 533, 110–114 (2016). 27147029 10.1038/nature17947 PMC4883664

[3] Yi T., Cyster J. G., EBI2-mediated bridging channel positioning supports splenic dendritic cell homeostasis and particulate antigen capture. Elife 2, e00757 (2013). 23682316 10.7554/eLife.00757 PMC3654440

[4] Kelly L. M., Pereira J. P., Yi T., Xu Y., Cyster J. G., EBI2 guides serial movements of activated B cells and ligand activity is detectable in lymphoid and nonlymphoid tissues. J Immunol 187, 3026–3032 (2011). 21844396 10.4049/jimmunol.1101262 PMC3169736

[5] Emgård J. , Oxysterol Sensing through the Receptor GPR183 Promotes the Lymphoid-Tissue-Inducing Function of Innate Lymphoid Cells and Colonic Inflammation. Immunity 48, 120–132.e128 (2018). 29343433 10.1016/j.immuni.2017.11.020 PMC5772175

[6] Chu C. , Anti-microbial Functions of Group 3 Innate Lymphoid Cells in Gut-Associated Lymphoid Tissues Are Regulated by G-Protein-Coupled Receptor 183. Cell Rep 23, 3750–3758 (2018). 29949760 10.1016/j.celrep.2018.05.099 PMC6209103

[7] Chen K. Y. , Inflammation switches the chemoattractant requirements for naive lymphocyte entry into lymph nodes. Cell 188, 1019–1035.e1022 (2025). 39708807 10.1016/j.cell.2024.11.031 PMC11845304

[8] Ceglia S. , An epithelial cell-derived metabolite tunes immunoglobulin A secretion by gut-resident plasma cells. Nat Immunol 24, 531–544 (2023). 36658240 10.1038/s41590-022-01413-w PMC10243503

[9] Frascoli M. , Skin γ*Δ* T cell inflammatory responses are hardwired in the thymus by oxysterol sensing via GPR183 and calibrated by dietary cholesterol. Immunity 56, 562–575.e566 (2023). 36842431 10.1016/j.immuni.2023.01.025 PMC10306310

[10] Wanke F. , EBI2 Is Highly Expressed in Multiple Sclerosis Lesions and Promotes Early CNS Migration of Encephalitogenic CD4 T Cells. Cell Rep 18, 1270–1284 (2017). 28147280 10.1016/j.celrep.2017.01.020

[11] Yi T. , Oxysterol gradient generation by lymphoid stromal cells guides activated B cell movement during humoral responses. Immunity 37, 535–548 (2012). 22999953 10.1016/j.immuni.2012.06.015 PMC3465460

[12] Hannedouche S. , Oxysterols direct immune cell migration via EBI2. Nature 475, 524–527 (2011). 21796212 10.1038/nature10280 PMC4297623

[13] Liu C. , Oxysterols direct B-cell migration through EBI2. Nature 475, 519–523 (2011). 21796211 10.1038/nature10226

[14] Cyster J. G., Dang E. V., Reboldi A., Yi T., 25-Hydroxycholesterols in innate and adaptive immunity. Nature Reviews Immunology 14, 731–743 (2014). 10.1038/nri3755 25324126

[15] Dang E. V., Reboldi A., Cholesterol sensing and metabolic adaptation in tissue immunity. Trends Immunol 45, 861–870 (2024). 39424470 10.1016/j.it.2024.09.013 PMC11560508

[16] Baptista A. P. , The Chemoattractant Receptor Ebi2 Drives Intranodal Naive CD4+ T Cell Peripheralization to Promote Effective Adaptive Immunity. Immunity 50, 1188–1201.e1186 (2019). 31053504 10.1016/j.immuni.2019.04.001

[17] Hannedouche S. , Oxysterols direct immune cell migration via EBI2. Nature 475, 524–527 (2011). 21796212 10.1038/nature10280 PMC4297623

[18] Li J., Lu E., Yi T., Cyster J. G., EBI2 augments Tfh cell fate by promoting interaction with IL-2-quenching dendritic cells. Nature 533, 110–114 (2016). 27147029 10.1038/nature17947 PMC4883664

[19] Rodda L. B. , Single-Cell RNA Sequencing of Lymph Node Stromal Cells Reveals Niche-Associated Heterogeneity. Immunity 48, 1014–1028.e1016 (2018). 29752062 10.1016/j.immuni.2018.04.006 PMC5971117

[20] Gundra U. M. , Alternatively activated macrophages derived from monocytes and tissue macrophages are phenotypically and functionally distinct. Blood 123, e110–e122 (2014). 24695852 10.1182/blood-2013-08-520619 PMC4023427

[21] Park K., Scott A. L., Cholesterol 25-hydroxylase production by dendritic cells and macrophages is regulated by type I interferons. J Leukoc Biol 88, 1081–1087 (2010). 20699362 10.1189/jlb.0610318 PMC2996899

[22] Bauman D. R. , 25-Hydroxycholesterol secreted by macrophages in response to Toll-like receptor activation suppresses immunoglobulin A production. Proc Natl Acad Sci U S A 106, 16764–16769 (2009). 19805370 10.1073/pnas.0909142106 PMC2757821

[23] Zou T., Garifulin O., Berland R., Boyartchuk V. L., Listeria monocytogenes infection induces prosurvival metabolic signaling in macrophages. Infect Immun 79, 1526–1535 (2011). 21263022 10.1128/IAI.01195-10 PMC3067555

[24] Xiao J. , 25-Hydroxycholesterol regulates lysosome AMP kinase activation and metabolic reprogramming to educate immunosuppressive macrophages. Immunity 57, 1087–1104.e1087 (2024). 38640930 10.1016/j.immuni.2024.03.021

[25] Dang E. V., McDonald J. G., Russell D. W., Cyster J. G., Oxysterol Restraint of Cholesterol Synthesis Prevents AIM2 Inflammasome Activation. Cell 171, 1057–1071.e1011 (2017). 29033131 10.1016/j.cell.2017.09.029 PMC5693620

[26] Reboldi A. , 25-Hydroxycholesterol suppresses interleukin-1–driven inflammation downstream of type I interferon. Science 345, 679–684 (2014). 25104388 10.1126/science.1254790 PMC4289637

[27] Gold E. S. , 25-Hydroxycholesterol acts as an amplifier of inflammatory signaling. Proceedings of the National Academy of Sciences 111, 10666–10671 (2014). 10.1073/pnas.1404271111 PMC4115544 24994901

[28] Liu S. Y. , Interferon-inducible cholesterol-25-hydroxylase broadly inhibits viral entry by production of 25-hydroxycholesterol. Immunity 38, 92–105 (2013). 23273844 10.1016/j.immuni.2012.11.005 PMC3698975

[29] Blanc M. , The Transcription Factor STAT-1 Couples Macrophage Synthesis of 25-Hydroxycholesterol to the Interferon Antiviral Response. Immunity 38, 106–118 (2013). 23273843 10.1016/j.immuni.2012.11.004 PMC3556782

[30] Li C. , 25-Hydroxycholesterol Protects Host against Zika Virus Infection and Its Associated Microcephaly in a Mouse Model. Immunity 46, 446–456 (2017). 28314593 10.1016/j.immuni.2017.02.012 PMC5957489

[31] Abrams M. E. , Oxysterols provide innate immunity to bacterial infection by mobilizing cell surface accessible cholesterol. Nature Microbiology 5, 929–942 (2020). 10.1038/s41564-020-0701-5 PMC7442315 32284563

[32] Zhou Q. D. , Interferon-mediated reprogramming of membrane cholesterol to evade bacterial toxins. Nature Immunology 21, 746–755 (2020). 32514064 10.1038/s41590-020-0695-4 PMC7778040

[33] Martinez F. O. , Genetic programs expressed in resting and IL-4 alternatively activated mouse and human macrophages: similarities and differences. Blood 121, e57–69 (2013). 23293084 10.1182/blood-2012-06-436212

[34] Reboldi A. , Inflammation. 25-Hydroxycholesterol suppresses interleukin-1-driven inflammation downstream of type I interferon. Science 345, 679–684 (2014). 25104388 10.1126/science.1254790 PMC4289637

[35] Blanc M. , The transcription factor STAT-1 couples macrophage synthesis of 25-hydroxycholesterol to the interferon antiviral response. Immunity 38, 106–118 (2013). 23273843 10.1016/j.immuni.2012.11.004 PMC3556782

[36] Wirth L. , High-Dimensional Analysis of Type 2 Lymphocyte Dynamics During Mepolizumab or Dupilumab Treatment in Severe Asthma. Allergy n/a. 10.1111/all.16633 PMC12444942 40566935

[37] Gieseck R. L., Wilson M. S., Wynn T. A., Type 2 immunity in tissue repair and fibrosis. Nature Reviews Immunology 18, 62–76 (2018). 10.1038/nri.2017.90 28853443

[38] Lloyd C. M., Snelgrove R. J., Type 2 immunity: Expanding our view. Science Immunology 3, eaat1604 (2018). 29980619 10.1126/sciimmunol.aat1604

[39] Spellberg B., Edwards J., Type 1/Type 2 Immunity in Infectious Diseases. Clinical infectious diseases : an official publication of the Infectious Diseases Society of America 32, 76–102 (2001). 11118387 10.1086/317537

[40] Zheng Y., Dang E. V., Novel mechanistic insights underlying fungal allergic inflammation. PLoS Pathog 19, e1011623 (2023). 37703276 10.1371/journal.ppat.1011623 PMC10499257

[41] Zheng Y. , Alternatively activated monocyte-derived myeloid cells promote extracellular pathogen persistence within pulmonary fungal granulomas. bioRxiv, 2025.2005.2023.655817 (2025). 10.1084/jem.20260208 PMC13154407 42008293

[42] Imai T. , Selective recruitment of CCR4-bearing Th2 cells toward antigen-presenting cells by the CC chemokines thymus and activation-regulated chemokine and macrophage-derived chemokine. International Immunology 11, 81–88 (1999). 10050676 10.1093/intimm/11.1.81

[43] Mikhak Z. , Contribution of CCR4 and CCR8 to antigen-specific T(H)2 cell trafficking in allergic pulmonary inflammation. J Allergy Clin Immunol 123, 67–73.e63 (2009). 19062085 10.1016/j.jaci.2008.09.049 PMC2782398

[44] Iliev I. D. , Focus on fungi. Cell 187, 5121–5127 (2024). 39303681 10.1016/j.cell.2024.08.016 PMC11722117

[45] Organization W. H., "WHO fungal priority pathogens list to guide research, development and public health action," (2022).

[46] Pereira J. P., Kelly L. M., Xu Y., Cyster J. G., EBI2 mediates B cell segregation between the outer and centre follicle. Nature 460, 1122–1126 (2009). 19597478 10.1038/nature08226 PMC2809436

[47] Gurram R. K. , Crosstalk between ILC2s and Th2 cells varies among mouse models. Cell Reports 42, (2023). 10.1016/j.celrep.2023.112073 PMC10394112 36735533

[48] Czimmerer Z. , The Transcription Factor STAT6 Mediates Direct Repression of Inflammatory Enhancers and Limits Activation of Alternatively Polarized Macrophages. Immunity 48, 75–90.e76 (2018). 29343442 10.1016/j.immuni.2017.12.010 PMC5772169

[49] Ohmori Y., Hamilton T. A., STAT6 is required for the anti-inflammatory activity of interleukin-4 in mouse peritoneal macrophages. J Biol Chem 273, 29202–29209 (1998). 9786931 10.1074/jbc.273.44.29202

[50] Dang E. V. , Secreted fungal virulence effector triggers allergic inflammation via TLR4. Nature 608, 161–167 (2022). 35896747 10.1038/s41586-022-05005-4 PMC9744105

[51] Bidault G. , SREBP1-induced fatty acid synthesis depletes macrophages antioxidant defences to promote their alternative activation. Nature Metabolism 3, 1150–1162 (2021). 10.1038/s42255-021-00440-5 PMC7611716 34531575

[52] Brown M. S., Goldstein J. L., The SREBP pathway: regulation of cholesterol metabolism by proteolysis of a membrane-bound transcription factor. Cell 89, 331–340 (1997). 9150132 10.1016/s0092-8674(00)80213-5

[53] Liu Z. , Fate Mapping via Ms4a3-Expression History Traces Monocyte-Derived Cells. Cell 178, 1509–1525.e1519 (2019). 31491389 10.1016/j.cell.2019.08.009

[54] Van den Bossche J. , Alternatively activated macrophages engage in homotypic and heterotypic interactions through IL-4 and polyamine-induced E-cadherin/catenin complexes. Blood 114, 4664–4674 (2009). 19726720 10.1182/blood-2009-05-221598

[55] Cronan M. R. , Macrophage Epithelial Reprogramming Underlies Mycobacterial Granuloma Formation and Promotes Infection. Immunity 45, 861–876 (2016). 27760340 10.1016/j.immuni.2016.09.014 PMC5268069

[56] Bohrer A. C. , Rapid GPR183-mediated recruitment of eosinophils to the lung after Mycobacterium tuberculosis infection. Cell Rep 40, 111144 (2022). 35905725 10.1016/j.celrep.2022.111144 PMC9460869

[57] Foo C. X. , GPR183 antagonism reduces macrophage infiltration in influenza and SARS-CoV-2 infection. Eur Respir J 61, (2023). 10.1183/13993003.01306-2022 PMC9686317 36396144

[58] Conlon T. M., Yildirim A., Oxysterol metabolism dictates macrophage influx during SARS-CoV-2 infection. Eur Respir J 61, (2023). 10.1183/13993003.02417-2022 36858446

[59] Ngo M. D. , A Blunted GPR183/Oxysterol Axis During Dysglycemia Results in Delayed Recruitment of Macrophages to the Lung During Mycobacterium tuberculosis Infection. J Infect Dis 225, 2219–2228 (2022). 35303091 10.1093/infdis/jiac102 PMC9200159

[60] Shen Z. J. , Epstein-Barr Virus-induced Gene 2 Mediates Allergen-induced Leukocyte Migration into Airways. Am J Respir Crit Care Med 195, 1576–1585 (2017). 28125291 10.1164/rccm.201608-1580OC PMC5476910

[61] Sato K. , Production of IL-17A at Innate Immune Phase Leads to Decreased Th1 Immune Response and Attenuated Host Defense against Infection with Cryptococcus deneoformans. J Immunol 205, 686–698 (2020). 32561568 10.4049/jimmunol.1901238

[62] Fu M. S., Kawakami K., Drummond R. A., Adoptive Transfer of Cryptococcus neoformans-Specific CD4 T-Cells to Study Anti-fungal Lymphocyte Responses In Vivo. Methods Mol Biol 2667, 99–112 (2023). 37145278 10.1007/978-1-0716-3199-7_7

[63] Rittershaus P. C. , Glucosylceramide synthase is an essential regulator of pathogenicity of Cryptococcus neoformans. J Clin Invest 116, 1651–1659 (2006). 16741577 10.1172/JCI27890 PMC1466548

[64] Zhou S. , Pathogenic mycobacterium upregulates cholesterol 25-hydroxylase to promote granuloma development via foam cell formation. iScience 27, 109204 (2024). 38420591 10.1016/j.isci.2024.109204 PMC10901098

[65] Zeng W. , Characterization of Anti-Interferon-γ Antibodies in HIV-Negative Patients Infected With Disseminated Talaromyces marneffei and Cryptococcosis. Open Forum Infect Dis 6, ofz208 (2019). 31660325 10.1093/ofid/ofz208 PMC6788342

[66] Davis M. J. , Inbred SJL mice recapitulate human resistance to Cryptococcus infection due to differential immune activation. mBio 14, e0212323 (2023). 37800917 10.1128/mbio.02123-23 PMC10653822

[67] Kawakami K. , Contribution of interferon-gamma in protecting mice during pulmonary and disseminated infection with Cryptococcus neoformans. FEMS Immunol Med Microbiol 13, 123–130 (1996). 8731020 10.1016/0928-8244(95)00093-3

[68] Chen G. H. , The gamma interferon receptor is required for the protective pulmonary inflammatory response to Cryptococcus neoformans. Infect Immun 73, 1788–1796 (2005). 15731080 10.1128/IAI.73.3.1788-1796.2005 PMC1064966

[69] Hardison S. E. , Pulmonary infection with an interferon-gamma-producing Cryptococcus neoformans strain results in classical macrophage activation and protection. Am J Pathol 176, 774–785 (2010). 20056835 10.2353/ajpath.2010.090634 PMC2808084

[70] Wang K. , Innate cells and STAT1-dependent signals orchestrate vaccine-induced protection against invasive Cryptococcus infection. mBio 15, e0194424 (2024). 39324785 10.1128/mbio.01944-24 PMC11481872

[71] Bohrer A. C. , Rapid GPR183-mediated recruitment of eosinophils to the lung after Mycobacterium tuberculosis infection. Cell Reports 40, 111144 (2022). 35905725 10.1016/j.celrep.2022.111144 PMC9460869

[72] Lavin Y. , Tissue-Resident Macrophage Enhancer Landscapes Are Shaped by the Local Microenvironment. Cell 159, 1312–1326 (2014). 25480296 10.1016/j.cell.2014.11.018 PMC4437213

[73] Zhang L. , GPR183 targets lung-resident CD301b&lt;sup&gt;+&lt;/sup&gt; conventional dendritic cells type 2 to a subtissular TSLP – TSLP receptor mediated survival niche within the adventital cuff. bioRxiv, 2022.2008.2028.505379 (2022).

[74] McCaffrey E. F. , The immunoregulatory landscape of human tuberculosis granulomas. Nature Immunology 23, 318–329 (2022). 35058616 10.1038/s41590-021-01121-x PMC8810384

[75] Pagán A. J., Ramakrishnan L., The Formation and Function of Granulomas. Annual Review of Immunology 36, 639–665 (2018). 10.1146/annurev-immunol-032712-100022 29400999

