# Supplementary material for: Spatial organization of pulmonary type 2 inflammation by a macrophage-derived cholesterol metabolite
Source: bioRxiv. 2025 Aug 1:2025.07.29.666625. Preprint. [Version 1] doi: 10.1101/2025.07.29.666625 (PMC12324256; doi:10.1101/2025.07.29.666625)
Supplement: Supplement 1 [file NIHPP2025.07.29.666625v1-supplement-1.pdf]

bioRxiv preprint doi: https://doi.org/10.1101/2025.07.29.666625; this version posted August 1, 2025. The copyright holder for this preprint (which was not certified by peer review) is the author/funder. This article is a US Government work. It is not subject to copyright under 17 USC 105 and is also made available for use under a CC0 license.

**Supplementary materials**

Materials and Methods

Figs. S1 to S5

Table S1

References

bioRxiv preprint doi: https://doi.org/10.1101/2025.07.29.666625; this version posted August 1, 2025. The copyright holder for this preprint (which was not certified by peer review) is the author/funder. This article is a US Government work. It is not subject to copyright under 17 USC 105 and is also made available for use under a CC0 license.

Supplementary Materials for

# Spatial organization of pulmonary type 2 inflammation by a macrophage-derived cholesterol metabolite

Yufan Zheng, Hannah E. Dobson, Makheni Jean Pierre, Lilly LeBlanc, Dominic P. Golec, Nathan Carrillo, Anshu Deewan, Claudia Rivera Cifuentes, Eduard Ansaldo, Eric V. Dang.

Corresponding author: eric.dang@nih.gov (E.V.D)

**The PDF file includes:**

Materials and Methods

Figs. S1 to S5

Table S1

References

bioRxiv preprint doi: https://doi.org/10.1101/2025.07.29.666625; this version posted August 1, 2025. The copyright holder for this preprint (which was not certified by peer review) is the author/funder. This article is a US Government work. It is not subject to copyright under 17 USC 105 and is also made available for use under a CC0 license.

## Materials and Methods

### Animal

All mouse experiments were approved by the National Institutes of Allergic and Infectious Diseases Animal Care and Use Committee (NIAID-ACUC) and were performed in accordance with NIAID-ACUC guidelines and under approved protocols (LHIM-4E). Female and male mus musculus were used in this study. In bone marrow chimera experiments, animals were irradiated at 6- to 8-week-old and analyzed 6-8 weeks following irradiation. Most other experiments were performed on adult animals between 8 and 20 weeks of age. *Gpr183*$^{Gfp/Gfp}$ (*1*) and *Ch25h*$^{tdTomato/+}$ (*2*) mice were gifted by Dr. Jason Cyster and Dr. Andrea Reboldi. *hCD2*$^{Cre}$*xGata3*$^{fl/fl}$ mice (line 20939) and *BoyJ* mice (8478) were ordered from NIAID-Taconic Exchange program. *Ch25h*$^{fl/fl}$ (JAX:037647) and *CD11c*$^{Cre}$ (JAX:008068) mice were purchased from Jackson Laboratory. All mice were housed in a specific pathogen–free environment.

### Bone marrow chimera

Mice were irradiated at 700 rads in two doses spaced 3hrs apart by 14DNR at NIAID. Bone marrow was prepared and injected the same day at least one hour after the final irradiation dose. Mice were monitored during 6-week reconstitution. Bleeding was performed to determine reconstitution efficiency.

### Flow cytometry

All mice for flow cytometry analysis in this study received retroorbital injection of 2 μg CD45 antibody 3 to 5 minutes before euthanasia to label intravascular cells. For lung samples, tissues were collected directly into 5 ml digestion buffer (HBSS with 1 mg/ml collagenase II (Gobio #17-101-015), 1 mg/ml Dispase (Gibco #17-105-041), and 10 μg/ml DNase I (Sigma-Aldrich

bioRxiv preprint doi: https://doi.org/10.1101/2025.07.29.666625; this version posted August 1, 2025. The copyright holder for this preprint (which was not certified by peer review) is the author/funder. This article is a US Government work. It is not subject to copyright under 17 USC 105 and is also made available for use under a CC0 license.

#11284932001) and then digested using a GentleMACS (Miltenyi Biotec). Digestion was stopped by adding 5 ml flow buffer (PBS with 2% FBS and 2 mM EDTA) on ice. Samples were immediately filtered using 70 μm strainers and centrifuged to remove digestion buffer. Red blood cells were removed by ACK lysis buffer (BioLegend #420302) for 5 min on ice and cells were then spun, washed, and aliquoted in 96-well round bottom plates for staining. Flow antibodies used in this study are listed in Table S1. Live/dead staining was performed for 10 min in PBS containing Fc Block before protein staining. Surface proteins were stained for 20 min on ice and then cells were acquired using a Cytek Aurora spectral flow cytometer. Intracellular and intranuclear proteins were stained with Transcription Factor Staining Buffer set (eBioscience #00-5523-00). Briefly, cells were fixed and permeabilized on ice for 1 hour and stained in permeabilization buffer containing antibodies for 1 hour at room temperature. Cells were washed and resuspended in flow buffer for acquisition. T cell restimulation was performed for cytokine analysis. Collected cells were cultured in RPMI 1640 culture medium with PMA (50 ng/ml) and ionomycin (500 ng/ml) for 4 hours and then processed for staining. Data were analyzed using FlowJo 10.10.0.

**T cell culture in vitro and competitive migration assay**

Spleens and Lymph nodes were harvested from mice and mashed through a 100 μm strainer in lymphocyte media (RPMI 1640 containing 10% FBS, 10mM HEPES, 2mM L-Glutamine, 55 μM 2-Mercaptoethanol, and antibiotics). CD4 T cells were isolated using EasySep™ Mouse CD4+ T Cell Isolation Kit (Stemcell technology #19852). T cells were resuspended in lymphocyte media at $6.67 \times 10^5$ cells/ml with an equal number of CD3/28 Dynabeads™ (ThermoFisher #11452D). 10 ng/ml IL-12 and 1 μg/ml IL-4 blocking antibodies were added to T helper 1 cell ($T_H1$s) culture and 20 ng/ml IL-4 and 1 μg/ml IFN-γ blocking antibodies were

bioRxiv preprint doi: https://doi.org/10.1101/2025.07.29.666625; this version posted August 1, 2025. The copyright holder for this preprint (which was not certified by peer review) is the author/funder. This article is a US Government work. It is not subject to copyright under 17 USC 105 and is also made available for use under a CC0 license.

added to T helper 2 cell ($T_H2$s) culture. T cells were left at 37°C with 5% $CO_2$ for 4 days before use in trans-well assays.

$T_H1$s and $T_H2$s were subsequently resuspended at 1 x $10^7$/ml in migration media (RPMI1640 with 0.5% fatty-acid free bovine serum albumin, penicillin/streptomyces, and 10mM HEPES buffer). 50 µl $T_H1$s and 50 µl $T_H2$s were carefully added to the top of each transwell insert at the same time. 10 µl of each were added to a well with 600 µl of media but no transwell insert as the input. The plate was then incubated at 37 °C and 5 % $CO_2$ for 3hrs with minimal disturbance. Afterwards, the transwell inserts were discarded and the cells were transferred to a 96-well round-bottom plate and resuspended in 200 µl of flow buffer. Cells from each well were acquired by flow cytometry for the same amount of time. The input % was calculated by dividing the cell numbers from the migration wells into 5 times cell number from the input well.

**Measurement for GPR183-dependent migration activity in lung tissues**

Lung samples were weighed and homogenized in 20 µl times the weight in mg with migration media. The lung homogenate was centrifuged at 300 rpm for 10 min to remove the cellular components first. The supernatant was then transferred to a new tube and centrifuged at 3000 rpm for 10 min to remove other debris. The supernatant was transferred to a new tube and stored at -80°C. M12 cells with 50 % GPR183-GFP transfected (*3*) (gifted by Dr. Jason Cyster) were cultured in Roswell Park Memorial Institute Medium, RPMI, supplemented with 10 % FBS, 10 mM HEPES buffer, penicillin/streptomyces, 2 mM L-Glutamine, and 55 µM 2-Mercaptoethanol at 37 °C and 5 % $CO_2$.

M12 cells were resuspended at 1 x $10^7$/ml in migration media. Lung extracts were diluted 1:10 in migration media. 600 µl of media, 10 nM 7 α, 25-HC, or diluted lung extracts were added to

bioRxiv preprint doi: https://doi.org/10.1101/2025.07.29.666625; this version posted August 1, 2025. The copyright holder for this preprint (which was not certified by peer review) is the author/funder. This article is a US Government work. It is not subject to copyright under 17 USC 105 and is also made available for use under a CC0 license.

wells of a 24-well plate in duplicate and 5 µm transwell inserts were placed on top. 100µl of EBI2-GFP M12 cells (1 x $10^6$) were carefully added to the top of each transwell insert. 20µl of EBI2-GFP M12 cells were added to a well with 600 µl of media but no transwell insert for the input. The plate was then incubated at 37 °C and 5 % $CO_2$ for 3 hours with minimal disturbance. Afterwards, the transwell inserts were discarded and the cells were transferred to a 96-well round-bottom plate and resuspended in 200µl of flow buffer for the same analysis by flow cytometry as mentioned above.

**RNA extraction and real-time quantitative PCR (RT-qPCR)**

The whole process was performed on ice or 4 °C. Lung samples were homogenized in TRIzol (Invitrogen #15596026) followed by adding 200 µl Chloroform. Samples were well-mixed and centrifuged at 12,900 g for 15 min. The equal volume of 2-propanol was mixed into the supernatant in a new tube and the samples were precipitated on ice for 10 min followed by 15 min centrifuge at 12,900 g. The supernatant was discarded. The precipitated pellets were washed by 75 % ethanol for twice and air-dried for 5 min. RNA samples were resuspended in nuclease-free water and stored at -80 °C or used for the reverse reaction with SuperScript™ IV Reverse Transcriptase (Thermo Fisher #18090010). Complementary DNA (cDNA) was stored at -20 °C or subsequently used for RT-qPCR with PowerTrack™ SYBR Green Master Mix (Thermo Fisher #A46110). *Ch25h* primers, forward: TGCATCACCAGAACTCGTCC, reverse: GGGAAGTCATAGCCCGAGTG. *Cyp7b1* primer, forward: GGAGCCACGACCCTAGATG, reverse: TGCCAAGATAAGGAAGCCAAC. *Cyp46a1* primer: *Cyp27a1* primer, forward: ATCTGGGTTGGGAAGGTG, reverse: CATTGCTCTCCTTGTGCGATG. The level of mRNA expression was all relative to *Rplp0* (also known as 36B4) expression. *Rplp0* primers, forward: GGGCATCACCACGAAAATCTC, reverse: CTGCCGTTGTCAAACACCT.

bioRxiv preprint doi: https://doi.org/10.1101/2025.07.29.666625; this version posted August 1, 2025. The copyright holder for this preprint (which was not certified by peer review) is the author/funder. This article is a US Government work. It is not subject to copyright under 17 USC 105 and is also made available for use under a CC0 license.

**Bone marrow-derived macrophage (BMDM) generation and culture**

Bone marrow was flushed out from mouse femurs and tibias and filtered through 70 μm into DMEM medium (10% FBS, 10 mM HEPES buffer, penicillin/streptomyces, 2 mM L-Glutamine). M-CSF was produced by M-CSF-NIH3T3 cell line and added into DMDM medium (10%) to support macrophage differentiation. BMDMs were harvested 7 days after differentiation.

**Thioglycolate-elicited peritoneal macrophage (PEM) collection**

Thioglycolate (3%) was injected intraperitoneally 4 days before the collection. 10 ml PBS was injected into peritoneal cavity to collect PEMs. PEMs were maintained in DMEM medium (10% FBS, 10 mM HEPES buffer, penicillin/streptomyces, 2 mM L-Glutamine).

**Single-cell RNA sequencing (scRNA-seq) for *Cryptococcus neoformans (Cn)* infection**

Mice at different time points were infected separately and harvested at the same time. Single cell suspension was made in the same way as flow cytometry experiments. Hashtag antibodies (TotalSeq™-C0301 to C315 anti-mouse Hashtag 1 to 15 Antibody, Biolegend #155861 to #155889) were added into the staining cocktail. Macrophages (Live/CD45$^+$/IV$^-$/B220$^-$/CD90.2$^-$/Ly6G$^-$/Ly6C$^-$/MertK$^+$/CD64$^+$) were sorted from these samples and pooled together for generating library. The library was prepared based on Chromium Next GEM Single Cell 5' Reagent Kits v2 (10X Genomics, document number: CG000220 Rev F). The sequencing was done at Center for Human Immunology/NIAID with NextSeq 1000/2000 P2 XLEAP-SBS Reagent Kit (Illumina #20100987). The parameters were Read 1/Index 1/Index 2/Read 2: 27 / 10 / 10 / 91 cycles. The 10X CellRanger (v7.1.0) mkfastq and count pipelines were respectively used to generate FASTQ files and count matrices with the mm10 reference (refdata-gex-mm10-

bioRxiv preprint doi: https://doi.org/10.1101/2025.07.29.666625; this version posted August 1, 2025. The copyright holder for this preprint (which was not certified by peer review) is the author/funder. This article is a US Government work. It is not subject to copyright under 17 USC 105 and is also made available for use under a CC0 license.

2020-A). Downstream analysis was performed in R (v4.3.0) using Seurat (v4.3.0) (*4*). Libraries were filtered by applying thresholds based on log-transformed values. Specifically, cells with low total RNA content or a low number of detected genes—both below the 3 median absolute deviations (MAD) lower threshold—were excluded. Additionally, cells with high mitochondrial gene expression levels, high total RNA, high number of cells or high HTO counts, exceeding the 3 MAD upper threshold, were also excluded. After merging the samples using Seurat's inbuilt merge function, hashtags were demultiplexed using HTODemux from Seurat. Doublets and negatives were removed from further analysis, along with the hashtag HTO-10 (Day10-rep1), which was identified as an outlier based on low expression levels after demultiplexing. Dimensional reduction of the merged log10 normalized data was performed using 30 principal components and visualized using Uniform Manifold Approximation and Projections (UMAP). Unsupervised clustering was performed with a resolution of 0.7. Cell annotation was performed using SingleR (v2.2.0) (*5*) and mouse expression data (MouseRNAseqData) (*6*) from Celldex (v1.10.1). Cells labeled macrophages from the main cell types were subset for additional processing. Visualization was performed using Seurat's visualization tools and ggplot2.

**GPR183 bone marrow overexpression**

On day 1, donor mice were injected with 150 mg/kg 5-fluorouracil (5-FU) intraperitoneally. On day 4, PLAT-E cells were transfected with MSCV-IRES-*Gfp* or MSCV-*Gpr183*-IRES-*Gfp* vectors to produce mouse-tropic retrovirus. On day 5, bone marrow was collected from the 5-FU-treated donor mice and plated into 24 well-plates with bone marrow culture medium (DMEM with 15 % FBS, 20 ng/mL IL-3, 50 ng/mL IL-6, 100 ng/mL SCF, Pen/Strep, HEPES). On day 6, supernatant was collected from transfected PLAT-E cells and filtered through a 0.45 µm syringe filter. Filtered virus was complemented with 4 µg/ml polybrene and HEPES. Bone

bioRxiv preprint doi: https://doi.org/10.1101/2025.07.29.666625; this version posted August 1, 2025. The copyright holder for this preprint (which was not certified by peer review) is the author/funder. This article is a US Government work. It is not subject to copyright under 17 USC 105 and is also made available for use under a CC0 license.

marrow cells were then treated with virus and centrifuged at room temperature for 2 hours at 2450 rpm. Bone marrow cells were recovered for 24 hours followed by the second spin-infection and another 24 hours recovery. On day 8, bone marrow cells were collected and washed for transfer into irradiated recipients.

**Thick-tissue imaging**

Mice were euthanized and perfused with 20 ml PBS followed by 20 ml 4 % PFA. Inflation with 2 % low-melting agarose was then performed for thick tissue sectioning using a Vibrating Blade Microtome. 500 µm sections were made and processed with Ce3D Tissue Clearing buffer set (*7*) (BioLegend #427702) according to commercial instructions. Briefly, sections were permeabilized at room temperature for 2 days with gentle shaking followed by antibody staining for another 2 days with gentle shaking. Sections were washed 3 times by washing buffer during next 24 hours and then cleared by clearing solution for overnight. Cleared sections were mounted with clearing solution in 4 spacers (120 µm thickness for each) on slides for imaging by Leica SP8 conjugated with 690 nm laser at Bioimaging Research Technologies Branch at NIAID.

**scRNA-seq for *Cn gcs1Δ* infection**

Myeloid cells (Live/CD45$^+$/IV$^-$/B220$^-$/CD90.2$^-$/Ly-6G$^-$/MHCII$^+$/CD11c$^+$/MHCII$^+$/CD64$^+$) were sorted at 35 days post-infection. Cells were also labeled by Hashtag antibodies and then pooled for library preparation and sequencing followed the same protocol as mentioned above. Data analysis was performed similarly, and no sample was excluded due to quality issue.

**RNA scope**

ACD RNAscope Multiplex Fluorescent V2 assays was used for detection of *Ch25h* and *Cyp7b1* RNA expression and co-detection of CD11c protein expression. Briefly, mice were euthanized

bioRxiv preprint doi: https://doi.org/10.1101/2025.07.29.666625; this version posted August 1, 2025. The copyright holder for this preprint (which was not certified by peer review) is the author/funder. This article is a US Government work. It is not subject to copyright under 17 USC 105 and is also made available for use under a CC0 license.

and perfused with 20 ml PBS followed by 20 ml 4 % PFA. OCT was used to inflate lungs through the trachea, and the tissues were flash frozen in molds and sectioned into 8 μm slices. Sections were fixed with 4% PFA and dehydrated for hydrogen peroxide incubation. Sections were then rehydrated for CD11c staining overnight and subsequently crosslinked with 10% neutral buffered formalin. Hybridization of both probes in different channels following the protease treatment was performed, and signal development for two channels was done separately with two different fluorophores. DAPI staining was performed 30 sec before mounting.

bioRxiv preprint doi: https://doi.org/10.1101/2025.07.29.666625; this version posted August 1, 2025. The copyright holder for this preprint (which was not certified by peer review) is the author/funder. This article is a US Government work. It is not subject to copyright under 17 USC 105 and is also made available for use under a CC0 license.

**fig. S1**

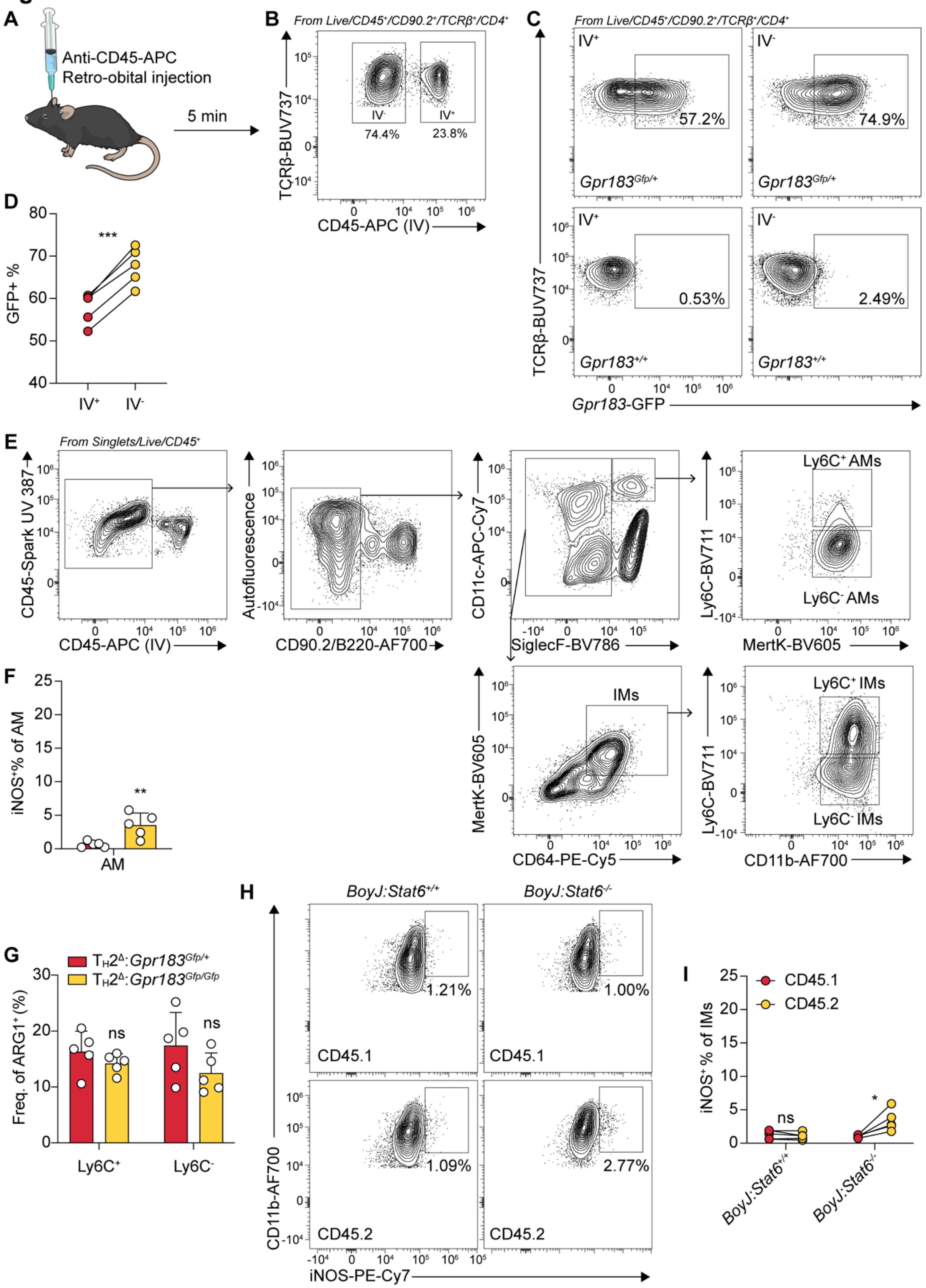


809

bioRxiv preprint doi: https://doi.org/10.1101/2025.07.29.666625; this version posted August 1, 2025. The copyright holder for this preprint (which was not certified by peer review) is the author/funder. This article is a US Government work. It is not subject to copyright under 17 USC 105 and is also made available for use under a CC0 license.

**Fig. S1. $T_H2$ sensing of oxysterols suppress iNOS expression in Ly6C+ macrophages.**

(**A**) Schematic of intravenous (IV) CD45 labeling.

(**B**) Representative flow cytometry plot showing IV labeling within the $CD4^+$ T cell compartment from *Cn*-infected mouse lung tissues at 10 dpi.

(**C**) Representative flow cytometry plots for *Gpr183*-GFP expression in $IV^+$ and $IV^-$ $CD4^+$ T cells from *Cn*-infected mouse lung tissues at 10 dpi.

(**D**) Quantification of *Gpr183*-$GFP^+$ portion in $IV^+$ versus $IV^-$ $CD4^+$ T cells from *Cn*-infected mouse lung tissues at 10 dpi; lines connect paired populations from the same host. $^{***}P < 0.001$, paired two-tailed Student's t-test.

(**E**) Gating strategy for lung resident $Ly6C^+$ or $Ly6C^-$ alveolar macrophages (AMs) and $Ly6C^-$ interstitial macrophages (IMs).

(**F**) Frequency of $iNOS^+$ cells in lung AMs from *Cn*-infected $Gpr183^{TH2\Delta}$ mice and their controls at 10 dpi.

(**G**) Frequency of $ARG1^+$ cells in lung $CD64^+$ macrophages from *Cn*-infected $Gpr183^{TH2\Delta}$ mice and their controls at 10 dpi.

(**H**) Representative flow cytometry plots of iNOS expression in $CD64^+$ lung macrophages from $CD45.1^+$ or $CD45.2^+$ donors in STAT6-knockout bone marrow mixed chimera at 10 dpi.

(**I**) Frequency of iNOS+ cells in lung CD64+ macrophages from $CD45.1^+$ or $CD45.2^+$ donors in STAT6-knockout bone marrow mixed chimera at 10 dpi.

bioRxiv preprint doi: https://doi.org/10.1101/2025.07.29.666625; this version posted August 1, 2025. The copyright holder for this preprint (which was not certified by peer review) is the author/funder. This article is a US Government work. It is not subject to copyright under 17 USC 105 and is also made available for use under a CC0 license.

fig. S2

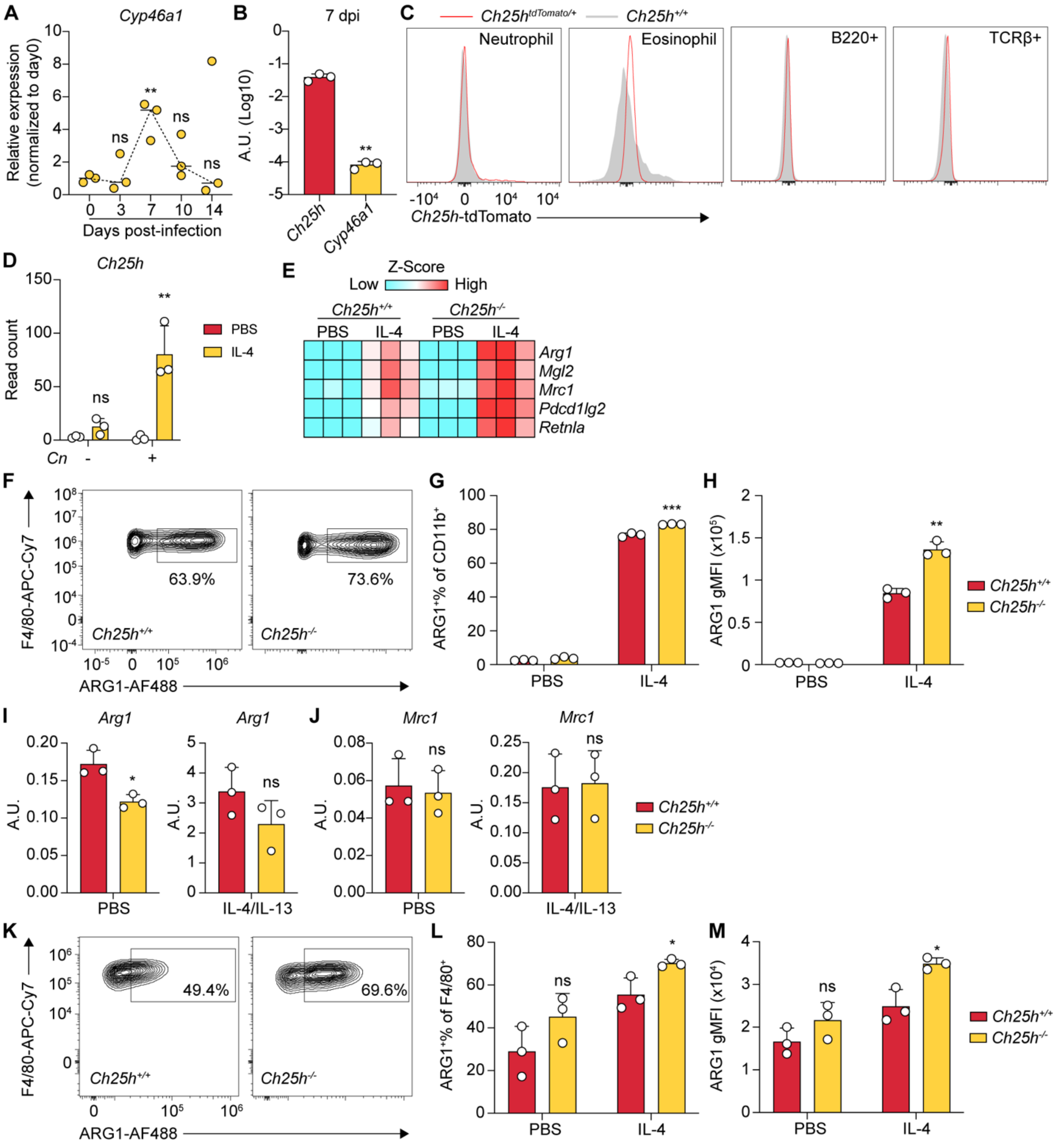


**Fig. S2. Lung macrophages establish GPR183 chemotactic gradients via *Ch25h* induction.**

(**A**) Relative mRNA levels of *Cyp46a1* in lung tissues over time following infection. Expression values were normalized to day 0 to show fold induction. $^{**}P < 0.01$ and ns, not significant, compared to day 0 by one-way ANOVA.

bioRxiv preprint doi: https://doi.org/10.1101/2025.07.29.666625; this version posted August 1, 2025. The copyright holder for this preprint (which was not certified by peer review) is the author/funder. This article is a US Government work. It is not subject to copyright under 17 USC 105 and is also made available for use under a CC0 license.

(**B**) Relative mRNA levels of *Ch25h* and *Cyp46a1* to *Rplp0* in lung tissues from *Cn*-infected mice at 7 dpi. A.U., arbitrary units. $^{**}P < 0.01$, unpaired two-tailed Student's t-test.

(**C**) Representative histograms of *Ch25h*-tdTomato expression across indicated cell populations. All these populations were gated from Live/CD45$^{+}$/IV$^{-}$. Neutrophils were gated by Ly-6G$^{+}$CD11b$^{+}$. Eosinophils were gated by SiglecF$^{+}$CD11b$^{+}$SSC-A$^{hi}$.

(D) Read count of *Ch25h* mRNA by bulk RNA-sequencing on bone marrow-derived macrophages (BMDMs) treated by indicated conditions. IL-4, 20 ng/ml; *Cn*, MOI=1. $^{**}P < 0.01$; ns, not significant; unpaired two-tailed Student's t-test.

(E) Heatmap of mRNA expression level of indicated markers for alternative activation of macrophages by bulk RNA-sequencing in BMDMs. Expression level was presented as Z-score. IL-4, 20 ng/ml.

(F) Representative flow cytometry plot showing ARG1 expression in *Ch25h*$^{+/+}$ versus *Ch25h*$^{-/-}$ BMDMs after 24 hours-IL-4 treatment. IL-4, 20 ng/ml.

(G) Frequency of ARG1$^{+}$ in *Ch25h*$^{+/+}$ versus *Ch25h*$^{-/-}$ BMDMs after 24 hours-IL-4 or PBS treatment. $^{***}P < 0.001$, unpaired two-tailed Student's t-test.

(H) Geometric mean fluorescence intensity (gMFI) of ARG1 in *Ch25h*$^{+/+}$ versus *Ch25h*$^{-/-}$ BMDMs after 24 hours-IL-4 or PBS treatment. $^{**}P < 0.01$, unpaired two-tailed Student's t-test.

(I) Relative mRNA levels of *Arg1* to *Rplp0* in thioglycolate-elicited peritoneal macrophages (PEMs) from *Ch25h*$^{+/+}$ versus *Ch25h*$^{-/-}$ mice after 24 hours-IL-4/IL-13 or PBS treatment. IL-4, 20 ng/ml; IL-13, 20 ng/ml. $^{*}P < 0.05$, ns, not significant, unpaired two-tailed Student's t-test.

bioRxiv preprint doi: https://doi.org/10.1101/2025.07.29.666625; this version posted August 1, 2025. The copyright holder for this preprint (which was not certified by peer review) is the author/funder. This article is a US Government work. It is not subject to copyright under 17 USC 105 and is also made available for use under a CC0 license.

(J) Relative mRNA levels of *Mrc1* to *Rplp0* in PEMs from *Ch25h*$^{+/+}$ versus *Ch25h*$^{-/-}$ mice after 24 hours-IL-4/IL-13 or PBS treatment. ns, not significant; unpaired two-tailed Student’s t-test.

(K) Representative flow cytometry plot showing ARG1 expression in *Ch25h*$^{+/+}$ versus *Ch25h*$^{-/-}$ PEMs after 24 hours-IL-4 treatment. IL-4, 20 ng/ml.

(L) Quantification of ARG1$^{+}$ frequency in *Ch25h*$^{+/+}$ versus *Ch25h*$^{-/-}$ PEMs after 24 hours-IL-4 or PBS treatment. $^{*}P < 0.05$, ns, not significant, unpaired two-tailed Student’s t-test.

(M) Quantification of ARG1 gMFI in *Ch25h*$^{+/+}$ versus *Ch25h*$^{-/-}$ PEMs after 24 hours-IL-4 or PBS treatment. $^{*}P < 0.05$, ns, not significant, unpaired two-tailed Student’s t-test.

bioRxiv preprint doi: https://doi.org/10.1101/2025.07.29.666625; this version posted August 1, 2025. The copyright holder for this preprint (which was not certified by peer review) is the author/funder. This article is a US Government work. It is not subject to copyright under 17 USC 105 and is also made available for use under a CC0 license.

fig. S3

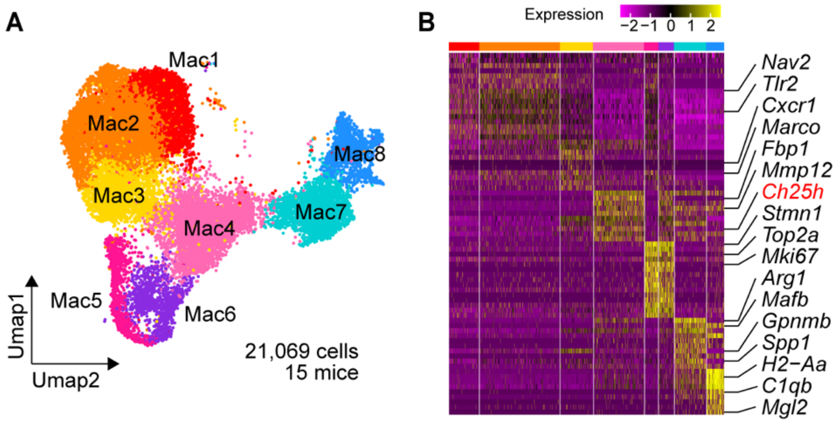


**Fig. S3. Single cell RNA-sequencing on $Ly6C^-$ lung macrophages.**

(**A**) UMAP visualization of original macrophage clusters from combined scRNA-seq replicates.

(**B**) Heatmap of top 10 differential expressed genes in each cluster.

bioRxiv preprint doi: https://doi.org/10.1101/2025.07.29.666625; this version posted August 1, 2025. The copyright holder for this preprint (which was not certified by peer review) is the author/funder. This article is a US Government work. It is not subject to copyright under 17 USC 105 and is also made available for use under a CC0 license.

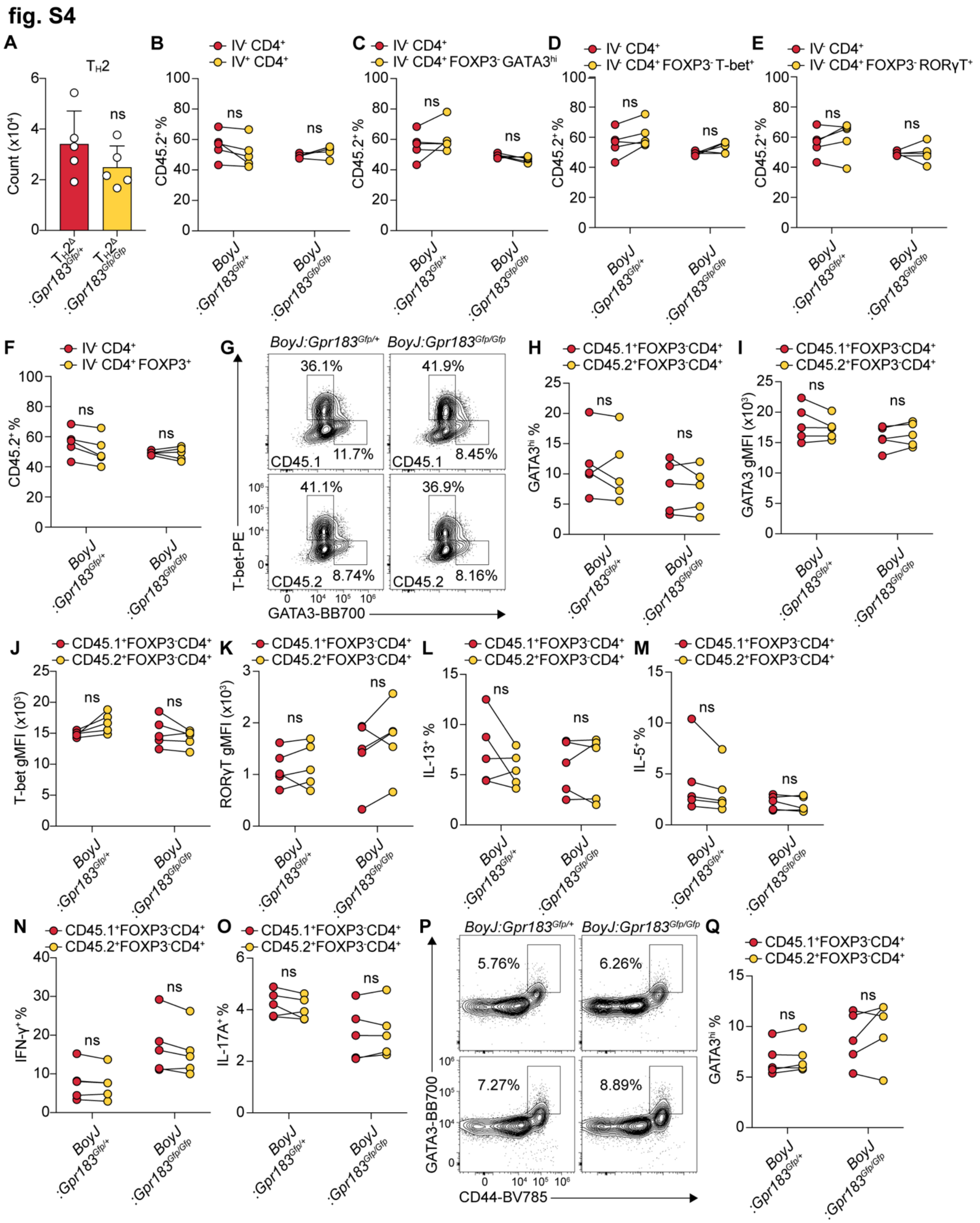


**Fig. S4. GPR183 is dispensable for T cell recruitment or $T_H2$ differentiation.**

bioRxiv preprint doi: https://doi.org/10.1101/2025.07.29.666625; this version posted August 1, 2025. The copyright holder for this preprint (which was not certified by peer review) is the author/funder. This article is a US Government work. It is not subject to copyright under 17 USC 105 and is also made available for use under a CC0 license.

(**A**) Quantification of lung $T_H2$ cells in *Cn*-infected *Gpr183*$^{TH2\Delta}$ mice and their controls at 10 dpi. Lung $T_H2$ cells were gated by Live/CD45$^+$/IV$^-$/CD90.2$^+$/TCRb$^+$/CD4$^+$/FOXP3$^-$/T-bet$^-$/GATA3$^{hi}$.

(**B**) Proportional contribution of CD45.2$^+$ cells to IV$^+$ versus IV$^-$CD4$^+$ T cells in lungs from *Cn*-infected mice with GPR183-knockout bone marrow mixed chimera and their controls at 10 dpi. ns, not significant by paired two-tailed Student's t-test.

(**C**) Proportional contribution of CD45.2$^+$ cells to total IV$^-$ CD4$^+$ T cells versus IV$^-$ CD4$^+$ FOXP3$^-$ GATA3$^{hi}$ $T_H2$ subset in lungs from *Cn*-infected mice with GPR183-knockout bone marrow mixed chimera and their controls at 10 dpi. ns, not significant by paired two-tailed Student's t-test.

(**D**) Proportional contribution of CD45.2$^+$ cells to IV$^-$ CD4$^+$ T cells and IV$^-$ CD4$^+$ FOXP3$^-$T-bet$^+$ $T_H1$ cells from *Cn*-infected mice with GPR183-knockout bone marrow mixed chimera and their controls at 10 dpi. ns, not significant by paired two-tailed Student's t-test.

(**E**) Proportional contribution of CD45.2$^+$ cells to IV$^-$ CD4$^+$ T cells and IV$^-$ CD4$^+$ FOXP3$^-$ ROR$\gamma$T$^+$ $T_H17$ cells from *Cn*-infected mice with GPR183-knockout bone marrow mixed chimera and their controls at 10 dpi. ns, not significant by paired two-tailed Student's t-test.

(**F**) Proportional contribution of CD45.2$^+$ cells to IV$^-$ CD4$^+$ T cells and IV$^-$ CD4$^+$ FOXP3$^+$ $T_{reg}$ cells from *Cn*-infected mice with GPR183-knockout bone marrow mixed chimera and their controls at 10 dpi. ns, not significant by paired two-tailed Student's t-test.

(**G**) Representative flow cytometry plots of T-bet and GATA3 expression in CD45.1$^+$ and CD45.2$^+$ FOXP3$^-$CD4$^+$ T cell population in the lungs from *Cn*-infected mice with GPR183-knockout bone marrow mixed chimera and their controls at 10 dpi. Gating: Singlets/Live/CD45$^+$/IV$^-$/CD90.2$^+$/TCR$^+$/CD4$^+$/FOXP3$^-$.

bioRxiv preprint doi: https://doi.org/10.1101/2025.07.29.666625; this version posted August 1, 2025. The copyright holder for this preprint (which was not certified by peer review) is the author/funder. This article is a US Government work. It is not subject to copyright under 17 USC 105 and is also made available for use under a CC0 license.

(**H**) Frequency of $GATA3^{hi}$ cells within $CD45.1^+$ or $CD45.2^+$ $FOXP3^-$ $CD4^+$ T cells in lungs from *Cn*-infected mice with GPR183-knockout bone marrow mixed chimera and their controls at 10 dpi. ns, not significant by paired two-tailed Student's t-test.

(**I**) GATA3 gMFI in $CD45.1^+$ or $CD45.2^+$ $FOXP3^-$ $CD4^+$ T cells from lungs in *Cn*-infected mice with GPR183-knockout bone marrow mixed chimera and their controls at 10 dpi. ns, not significant by paired two-tailed Student's t-test.

(**J**) T-bet geometric mean of fluorescent intensity (gMFI) in $CD45.1^+$ or $CD45.2^+$ $FOXP3^-$ $CD4^+$ T cells from lungs in *Cn*-infected mice with GPR183-knockout bone marrow mixed chimera and their controls at 10 dpi. ns, not significant by paired two-tailed Student's t-test.

(**K**) RORγT gMFI in $CD45.1^+$ or $CD45.2^+$ $FOXP3^-$ $CD4^+$ T cells from lungs in *Cn*-infected mice with GPR183-knockout bone marrow mixed chimera and their controls at 10 dpi. ns, not significant by paired two-tailed Student's t-test.

(**L**) Frequency of IL-$13^+$ cells among $CD45.1^+$ and $CD45.2^+$ $FOXP3^-$ $CD4^+$ T cells in lungs from *Cn*-infected mice with GPR183-knockout bone marrow mixed chimera and their controls at 10 dpi. ns, not significant by paired two-tailed Student's t-test.

(**M**) Frequency of IL-$5^+$ cells among $CD45.1^+$ and $CD45.2^+$ $FOXP3^-$ $CD4^+$ T cells in lungs from *Cn*-infected mice with GPR183-knockout bone marrow mixed chimera and their controls at 10 dpi. ns, not significant by paired two-tailed Student's t-test.

(**N**) Frequency of $IFN\gamma^+$ cells among $CD45.1^+$ and $CD45.2^+$ $FOXP3^-$ $CD4^+$ T cells in lungs from *Cn*-infected mice with GPR183-knockout bone marrow mixed chimera and their controls at 10 dpi. ns, not significant by paired two-tailed Student's t-test.

bioRxiv preprint doi: https://doi.org/10.1101/2025.07.29.666625; this version posted August 1, 2025. The copyright holder for this preprint (which was not certified by peer review) is the author/funder. This article is a US Government work. It is not subject to copyright under 17 USC 105 and is also made available for use under a CC0 license.

(**O**) Frequency of IL-17$^{+}$ cells among CD45.1$^{+}$ and CD45.2$^{+}$ FOXP3$^{-}$ CD4$^{+}$ T cells in lungs from *Cn*-infected mice with GPR183-knockout bone marrow mixed chimera and their controls at 10 dpi. ns, not significant by paired two-tailed Student's t-test.

(**P**) Representative flow cytometry plots of GATA3 expression in mediastinal lymph node CD45.1$^{+}$ and CD45.2$^{+}$ FOXP3$^{-}$CD4$^{+}$ T cells from *Cn*-infected mice with GPR183-knockout bone marrow mixed chimera and their controls at 10 dpi. Gating: Singlets/Live/CD45$^{+}$/IV$^{-}$/CD90.2$^{+}$/TCR$^{+}$/CD4$^{+}$/FOXP3$^{-}$.

(**Q**) Frequency of GATA3$^{hi}$ cells within CD45.1$^{+}$ and CD45.2$^{+}$ FOXP3$^{-}$ CD4$^{+}$ T cells in mediastinal lymph nodes from *Cn*-infected mice with GPR183-knockout bone marrow mixed chimera and their controls at 10 dpi. ns, not significant by paired two-tailed Student's t-test.

bioRxiv preprint doi: https://doi.org/10.1101/2025.07.29.666625; this version posted August 1, 2025. The copyright holder for this preprint (which was not certified by peer review) is the author/funder. This article is a US Government work. It is not subject to copyright under 17 USC 105 and is also made available for use under a CC0 license.

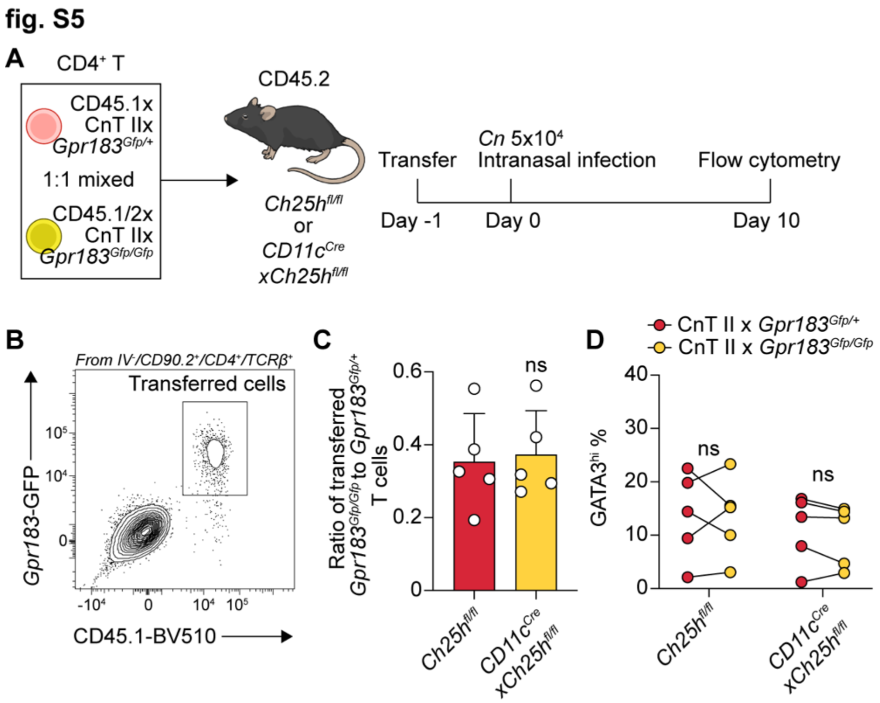


**Fig. S5. GPR183 is dispensable for cryptococcal antigen-specific T cell recruitment or $T_H2$ differentiation.**

(**A**) Schematic for adoptive T cell transfer experiment. Cryptococcal-specific TCR transgenic (CnT II) *Gpr183*$^{Gfp/+}$ or *GPR183*$^{Gfp/Gfp}$ CD4$^+$ T cells were transferred into *Ch25h*$^{fl/fl}$ or *CD11c*$^{Cre}$*xCh25h*$^{fl/fl}$ mice one day before infection (-1 dpi). Flow analysis was performed at 10 dpi.

(**B**) Representative flow cytometry plot for transferred *Gpr183*-GFP$^+$ CnT II T cells.

(**C**) Ratio of transferred CnT II *Gpr183*$^{Gfp/+}$ to *GPR183*$^{Gfp/Gfp}$ T cells in lungs of *Ch25h*$^{fl/fl}$ and *CD11c*$^{Cre}$x*Ch25h*$^{fl/fl}$ mice at 10 dpi. ns, not significant by unpaired two-tailed Student's t-test.

(**D**) Frequency of GATA3$^{hi}$ cells within transferred CnT II *Gpr183*$^{Gfp/+}$ or *GPR183*$^{Gfp/Gfp}$ FOXP3$^-$ CD4$^+$ T cells in lung tissues from *Cn*-infected *Ch25h*$^{fl/fl}$ or *CD11c*$^{Cre}$*xCh25h*$^{fl/fl}$ mice at 10 dpi. ns, not significant by paired two-tailed Student's t-test.

bioRxiv preprint doi: https://doi.org/10.1101/2025.07.29.666625; this version posted August 1, 2025. The copyright holder for this preprint (which was not certified by peer review) is the author/funder. This article is a US Government work. It is not subject to copyright under 17 USC 105 and is also made available for use under a CC0 license.

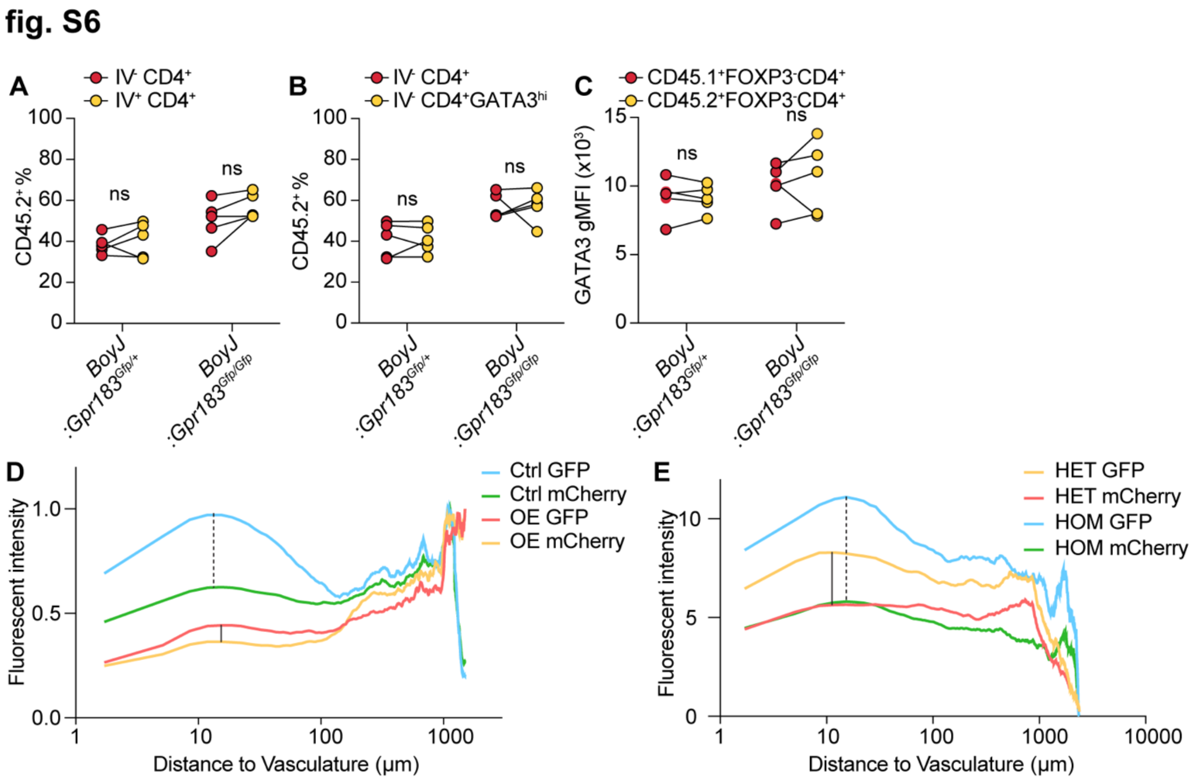


**Fig. S6. GPR183 guides TH2 intra-pulmonary positioning.**

(**A**) Proportional contribution of CD45.2$^+$ cells to IV$^-$ versus IV$^+$ CD4$^+$ T cells from *Cn gcs1Δ*-infected mice with GPR183-knockout bone marrow mixed chimera and their controls at 21 dpi.

(**B**) Proportional contribution of CD45.2$^+$ cells to total IV$^-$ CD4$^+$ T cells versus IV$^-$ CD4$^+$ FOXP3$^-$ GATA3$^{hi}$ T$_H$2 cells from *Cn gcs1Δ*-infected mice with GPR183-knockout bone marrow mixed chimera and their controls 21 dpi.

(**C**) GATA3 gMFI in CD45.1$^+$ or CD45.2$^+$ FOXP3$^-$ CD4$^+$ T cells from lungs in *Cn gcs1Δ*-infected mice with GPR183-knockout bone marrow mixed chimera and their controls 21 dpi.

(**D**) Quantification of fluorescent intensity at indicated distance to vasculature from images in Fig. 4A.

bioRxiv preprint doi: https://doi.org/10.1101/2025.07.29.666625; this version posted August 1, 2025. The copyright holder for this preprint (which was not certified by peer review) is the author/funder. This article is a US Government work. It is not subject to copyright under 17 USC 105 and is also made available for use under a CC0 license.

(**E**) Quantification of fluorescent intensity at indicated distance to vasculature from images in Fig. 4B.

bioRxiv preprint doi: https://doi.org/10.1101/2025.07.29.666625; this version posted August 1, 2025. The copyright holder for this preprint
(which was not certified by peer review) is the author/funder. This article is a US Government work. It is not subject to copyright under 17 USC 105 and is also made available for use under a CC0 license.

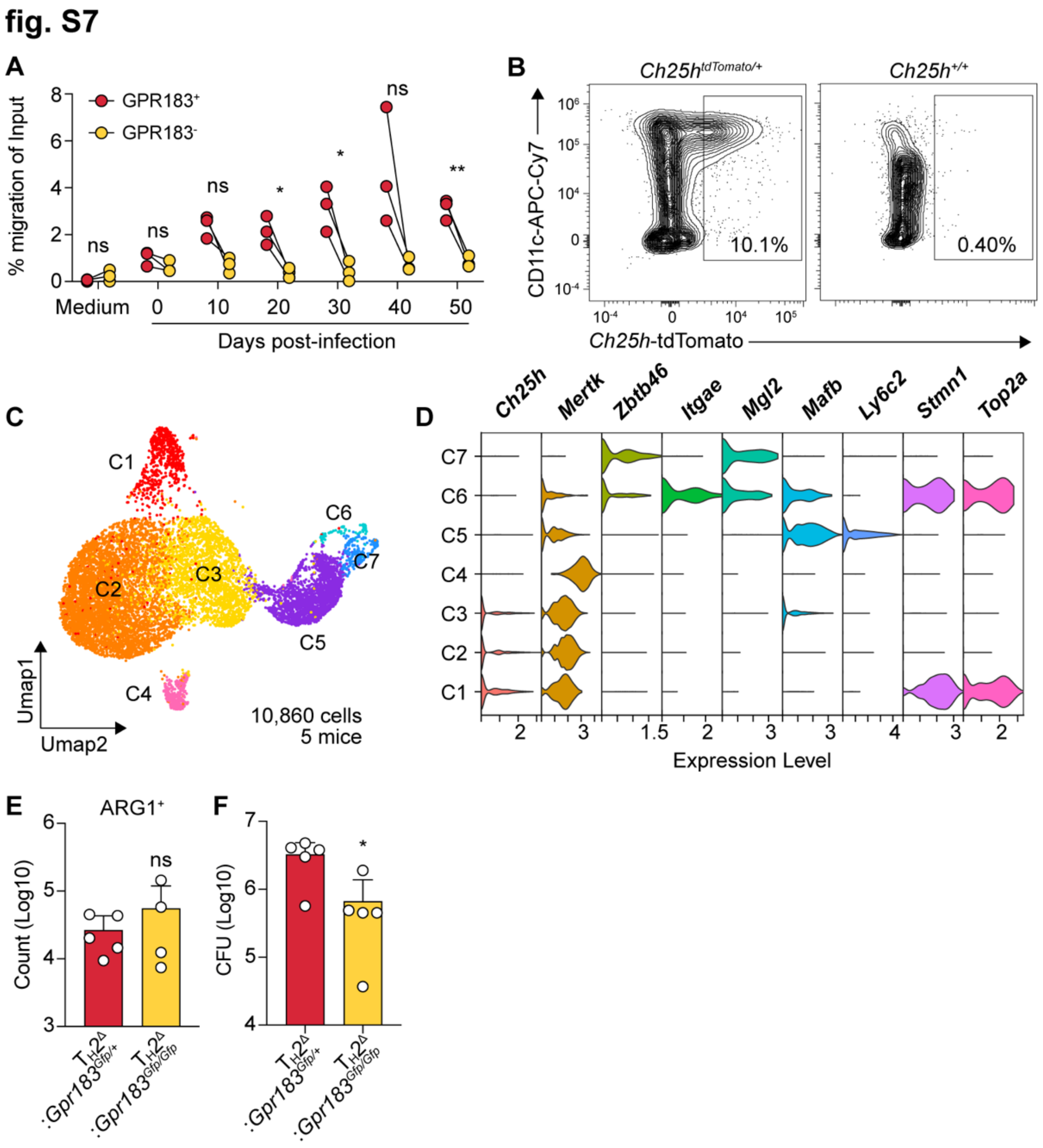


**Fig. S7. Granuloma-associated macrophages antagonize fungal clearance by positioning $T_H2$ cells.**

(**A**) Quantification of migration activity of GPR183$^+$ versus GPR183$^-$ M12 cells in response to lung extracts collected at indicated time points or migration media alone. $^*P < 0.05$, $^{**}P < 0.01$; ns, not significant; paired two-tailed Student's t-test.

bioRxiv preprint doi: https://doi.org/10.1101/2025.07.29.666625; this version posted August 1, 2025. The copyright holder for this preprint (which was not certified by peer review) is the author/funder. This article is a US Government work. It is not subject to copyright under 17 USC 105 and is also made available for use under a CC0 license.

(**B**) Representative flow cytometry plots of *Ch25h*-tdTomato expression in CD45$^+$ IV$^-$ immune cells at 35 dpi.

(**C**) UMAP visualization of original myeloid clusters from combined scRNA-seq replicates.

(D) Violin plots of the selected lineage marker expression across myeloid clusters.

(**E**) Frequency of ARG1$^+$ cells in lung CD64$^+$ macrophages from *Cn gcs1Δ*-infected *Gpr183*$^{TH2\Delta}$ mice and their controls at 21 dpi.

(**F**) Pulmonary fungal burden in *Cn gcs1Δ*-infected *Gpr183*$^{TH2\Delta}$ mice and their controls at 35 dpi. CFU, colony forming unit.

bioRxiv preprint doi: https://doi.org/10.1101/2025.07.29.666625; this version posted August 1, 2025. The copyright holder for this preprint (which was not certified by peer review) is the author/funder. This article is a US Government work. It is not subject to copyright under 17 USC 105 and is also made available for use under a CC0 license.

961 **Table S1**

| **Antibodies** | | |
|---|---|---|
| AF488 anti-mouse ARG1 (clone A1EXF5) | eBioscience | Cat# 53-3697-80 |
| AF488 anti-GFP (polyclonal) | ThermoFisher | Cat# A-21311 |
| AF647 anti-mouse Ly-6G (clone 1A8) | BioLegend | Cat# 127609 |
| AF700 anti-mouse EpCAM (clone G8.8) | BioLegend | Cat# 118240 |
| APC anti-mouse CD45 (clone 30-F11) | BioLegend | Cat# 103112 |
| APC-Cy7 anti-mouse CD11c (clone N418) | BioLegend | Cat# 117324 |
| APC-Cy anti-mouse IL-17A (clone TC11-18H10.1) | BioLegend | Cat# 506940 |
| BB700 anti-mouse GATA3 (clone L50823) | BD Bioscience | Cat# 566643 |
| BUV737 anti-mouse TCRβ (clone H57-597) | BD Bioscience | Cat# 612821 |
| BV421 anti-mouse CD3 (clone 17A2) | BioLegend | Cat# 100227 |
| BV421 anti-mouse IL-5 (clone TRFK5) | BioLegend | Cat# 504311 |
| BV421 anti-mouse Foxp3 (clone MF-14) | BioLegend | Cat# 126419 |
| BV421 anti-mouse PD-L2 (clone TY25) | BioLegend | Cat# 107219 |
| BV510 anti-mouse CD103 (clone 2E7) | BioLegend | Cat# 121423 |
| BV510 anti-mouse CD45.1 (clone A20) | BD Bioscience | Cat# 565278 |
| BV510 anti-mouse CD90.2 (clone 30-H12) | BioLegend | Cat# 105335 |
| BV605 anti-mouse CD4 (clone GK1.5) | BioLegend | Cat# 100451 |

bioRxiv preprint doi: https://doi.org/10.1101/2025.07.29.666625; this version posted August 1, 2025. The copyright holder for this preprint (which was not certified by peer review) is the author/funder. This article is a US Government work. It is not subject to copyright under 17 USC 105 and is also made available for use under a CC0 license.

| | | |
|---|---|---|
| BV605 anti-mouse MertK (clone 2B10C42) | BioLegend | Cat# 151517 |
| BV650 anti-mouse Ki67 (clone 11F6) | BioLegend | Cat# 151215 |
| BV711 anti-mouse Ly6C (clone HK1.4) | BioLegend | Cat# 128037 |
| BV711 anti-mouse IFNγ (clone XMG1.2) | BD Bioscience | Cat# 563736 |
| BV786 anti-mouse CD44 (clone IM7) | BD Bioscience | Cat# 563736 |
| BV786 anti-mouse SiglecF (clone E50-2440) | BD Bioscience | Cat# 740956 |
| FITC anti-mouse CD8 (clone S18018E) | BioLegend | Cat# 162313 |
| PE anti-mouse T-bet (clone 4B10) | BioLegend | Cat# 644809 |
| PE-Cy5 anti-mouse CD64 (clone X54-5/7.1) | BioLegend | Cat# 139332 |
| PE-Cy7 anti-mouse CD11c (clone N418) | BioLegend | Cat# 117317 |
| PE-Cy7 anti-mouse iNOS (clone CXNFT) | eBioscience | Cat# 25-5920-80 |
| PE-Cy7 anti-mouse Ly6G (clone 1A8) | BioLegend | Cat# 127617 |
| PE-Cy7 anti-mouse CD301b (MGL2) (clone URA-1) | BioLegend | Cat# 146807 |
| PE-Cy7 anti-mouse RORγt (clone B2D) | eBioscience | Cat# 25-6981-82 |
| PerCP-Cy5.5 anti-mouse MHCII (clone M5/114.15.2) | BioLegend | Cat# 107625 |
| R718 anti-mouse IL-13 (clone W19-895) | BD Bioscience | Cat# 569945 |
| Spark UV 387 anti-mouse CD45 (clone 30F-11) | BioLegend | Cat# 103188 |
| Spark UV 387 anti-mouse CD45.2 (clone 104) | BioLegend | Cat# 109868 |

962

bioRxiv preprint doi: https://doi.org/10.1101/2025.07.29.666625; this version posted August 1, 2025. The copyright holder for this preprint (which was not certified by peer review) is the author/funder. This article is a US Government work. It is not subject to copyright under 17 USC 105 and is also made available for use under a CC0 license.

## References

1. J. P. Pereira, L. M. Kelly, Y. Xu, J. G. Cyster, EBI2 mediates B cell segregation between the outer and centre follicle. *Nature* **460**, 1122-1126 (2009).
2. M. Frascoli *et al.*, Skin γδ T cell inflammatory responses are hardwired in the thymus by oxysterol sensing via GPR183 and calibrated by dietary cholesterol. *Immunity* **56**, 562-575.e566 (2023).
3. J. P. Pereira, L. M. Kelly, Y. Xu, J. G. Cyster, EBI2 mediates B cell segregation between the outer and centre follicle. *Nature* **460**, 1122-1126 (2009).
4. Y. Hao *et al.*, Integrated analysis of multimodal single-cell data. *Cell* **184**, 3573-3587.e3529 (2021).
5. D. Aran *et al.*, Reference-based analysis of lung single-cell sequencing reveals a transitional profibrotic macrophage. *Nature Immunology* **20**, 163-172 (2019).
6. B. A. Benayoun *et al.*, Remodeling of epigenome and transcriptome landscapes with aging in mice reveals widespread induction of inflammatory responses. *Genome Res* **29**, 697-709 (2019).
7. W. Li, R. N. Germain, M. Y. Gerner, High-dimensional cell-level analysis of tissues with Ce3D multiplex volume imaging. *Nature Protocols* **14**, 1708-1733 (2019).
